# The complex relationship between climate anomalies and reproductive attitudes and practices in low- and middle-income countries

**DOI:** 10.3389/fgwh.2025.1548648

**Published:** 2025-09-16

**Authors:** Meg L. Brown, Alex Severson, Sonia Tiedt, Aly Beeman, Nomi R. Fuchs, Theodora Gibbs

**Affiliations:** 1 Center for Population, Sustainability, and Health, School of Public Health, University of California, Berkeley, Berkeley, CA, United States; 2 Lumen Evaluation, Albuquerque, NM, United States; 3 Department of Political Science, University of New Mexico, Albuquerque, NM, United States; 4 School of Public Health, Imperial College London, London, United Kingdom; 5 YLabs, San Francisco, CA, United States; 6 YLabs, Kigali, Rwanda; 7 NRF Consulting Services, LLC, Seattle, WA, United States

**Keywords:** sexual and reproductive health and rights, climate change, contraception, gender, extreme heat, extreme rainfall

## Abstract

**Introduction:**

Climate change significantly impacts sexual and reproductive health (SRH) attitudes and practices, yet large-scale quantitative analyses exploring these effects are limited. This study investigates the historical associations between climate change, specifically temperature and precipitation anomalies, and key SRH attitudes and practices including contraception use, fertility preferences, and contraceptive autonomy.

**Methods:**

Using data from 74 IMPUMS-harmonized Demographic and Health Surveys merged with high-resolution climate data, we analyzed a sample of 820,746 non-pregnant, reproductive-aged women across 33 low- and middle-income countries from 2000 to 2016. Fixed-effect logistic regression models were employed to assess the association between climate anomalies and SRH attitudes and practices.

**Results:**

Pooled sample results indicate modest but significant associations globally: higher exposure to extreme heat in the year prior to survey administration was associated with lower odds of modern contraception use, lower odds of desire for children, and higher odds of contraceptive autonomy, while higher exposure to extreme precipitation was associated with lower odds of desire for children and higher odds of contraceptive autonomy. These associations were more pronounced when both temperature and precipitation anomalies occurred concurrently. Substantial demographic and geographic variability were observed, with mixed directionality and strength of association observed across countries and stronger associations observed among nulliparous women and younger respondents.

**Discussion:**

Our findings underscore the potential impact of climate change on SRH attitudes and practices, as well as SRH service delivery needs in the context of extreme heat and extreme precipitation, highlighting the importance of targeted, gender-responsive health interventions tailored to climate change-affected populations.

## 1 Introduction

Climate change poses a significant threat to health, both via direct impacts and indirect effects on social determinants of health, including infrastructure, economies, food and water access, health systems, and supply chains (1–8). These effects are expected to worsen in the coming years if comprehensive climate mitigation and adaptation actions are not taken, particularly within the low- and middle-income countries (LMICs) that are among the most vulnerable to climate change (6, 9–11). Moreover, in many contexts, women are especially susceptible to these adverse health effects as a result of persistent gender inequalities (12, 13). Yet both health and gender—along with the voices of those most affected by climate change—are often only marginally included in climate research and policymaking, or are left out altogether, resulting in the near or total absence of considerations of sexual and reproductive health (SRH) in climate adaptation research and policy and inhibiting progress towards more effective, transformative, and just climate action (14–20).

Access to SRH services is a critical aspect of realizing individuals' right to health, including ensuring that all people, including women and girls, have the ability to make decisions about their lives and their bodies freely and without coercion (21–23). As the impacts of climate change are increasingly being felt by individuals and communities, there is growing interest in understanding how climate change and climate-related extreme weather events may affect sexual and reproductive health and rights (SRHR) (19, 20, 24–27). Though research is limited and there is a need for greater methodological robustness in this area of investigation, experiences of climate change and climate-related processes such as extreme weather events—and their downstream effects on income, nutrition, water, and access to health services and products—have been linked with a range of adverse SRH outcomes, including impacts on gender based violence, maternal and newborn health, HIV, and others (20).

However, though it can be hypothesized that these downstream effects may in turn affect reproductive health and family planning needs, preferences, and service access, the impact of climate exposure on behaviors and preferences related to contraception and fertility has been significantly understudied (20). Somefun et al.'s recent analysis of drought exposure on contraception behavior and fertility preference in sub-Saharan Africa is the notable exception (28). This study found that drought had mixed effects on contraception use and desire for children at the country level, with some countries exhibiting higher odds of using contraception or wanting to delay or avoid childbirth and others exhibiting lower odds (28). Similarly, given that many contraceptive methods are heat-sensitive, with shelf stability temperature recommendations of 30° Celsius (29, 30), it is reasonable to hypothesize that in regions with unreliable electricity coverage and/or lack of appropriate climate controls, climate-related heatwaves and increases in ambient temperatures could result in contraceptive products being exposed to sustained temperatures above their shelf-stability thresholds. This exposure, in turn, could (1) compromise the effectiveness of these contraceptive methods and/or (2) result in reduced availability of contraceptive methods and increased waste if these products need to be discarded. However, there is limited data regarding how many contraceptive users might be impacted by exposure of their current contraceptive commodities to extreme heat and how elevated ambient temperatures might accelerate the degradation of the active pharmaceutical ingredients.

Climate change has the potential to significantly impact SRH, including contraception access, which would threaten decades of hard-won progress towards increasing FP access and improving SRH outcomes in LMICs. Backsliding on these gains could result in poorer health outcomes, reduced autonomy for women and girls, and greater gender inequity. Moreover, scholars have called for additional research at the nexus of climate change and SRH to address the above-described data gaps (19, 20, 24). The quantitative modeling results presented in this paper bridge this gap by examining the relationship between key climate-affected weather trends—temperature and precipitation anomalies—and SRH dynamics, including contraceptive use, contraceptive autonomy, and fertility preference. By using a rights-based approach to consider the timing, scope, and direction of these impacts, the research aims to contribute to foundational evidence critical for accelerating climate and SRH research and developing more effective strategies for ensuring the sexual and reproductive health and rights of all are fully realized even in a changing climate.

## 2 Materials and methods

### 2.1 Database construction

To construct the sample for this analysis, we merged health survey data with historic temperature and precipitation data. Temperature and precipitation were selected based on data availability and coverage across all countries of interest, to avoid replication of the work of others [e.g., (28)], and to ensure the analyses in this study could inform ongoing research using climate projection models to project future SRH impacts.

Health data were derived from Integrated Public Use Microdata Series (IPUMS)-harmonized Demographic and Health Surveys (DHS) (31). The DHS is a nationally representative household survey that provides comprehensive data on population, health, and nutrition indicators in developing countries, including modules for reproductive-age women (32). DHS procedures and survey questionnaires are reviewed and approved by ICF's Institutional Review Board (IRB) and, when appropriate, a host country IRB; all participation is voluntary and all participants provide informed consent (33). Households are surveyed in clusters, with approximately 25–30 households per cluster (34). The DHS program anonymizes survey respondents by using a numeric identifier and providing only cluster-level GPS coordinates, displacing urban coordinates by up to 2 km and rural coordinates by up to 5 km (with 1% displaced up to 10 km) (33). IPUMS DHS enables consistent analysis of DHS data across countries and time by harmonizing variables and linking survey documentation by variables (31).

Using the displaced coordinates for each respondent's cluster, DHS data were merged with high-resolution (0.05° × 0.05°) minimum and maximum daily temperature data from the University of California, Santa Barabara's Climate Hazards Center InfraRed Temperature with Stations (CHIRTS) database (35) and daily precipitation data from the Climate Hazards Center InfraRed Precipitation with Station (CHIRPS) database (36) for the 365 days prior to the survey administration date for each unique survey. Specifically, we analyzed the relationship between exposure to climate anomalies and reported contraception use, fertility preferences, and contraceptive autonomy among reproductive-age (i.e., 15–49 years old) women. The database also included socioeconomic and demographic variables, including age, education level, parity, marital status, wealth index, and urban/rural residence. Data were matched using Python following the guidance of Dorélien and Grace (37).

### 2.2 Eligibility criteria and sample

**Figure 1:**
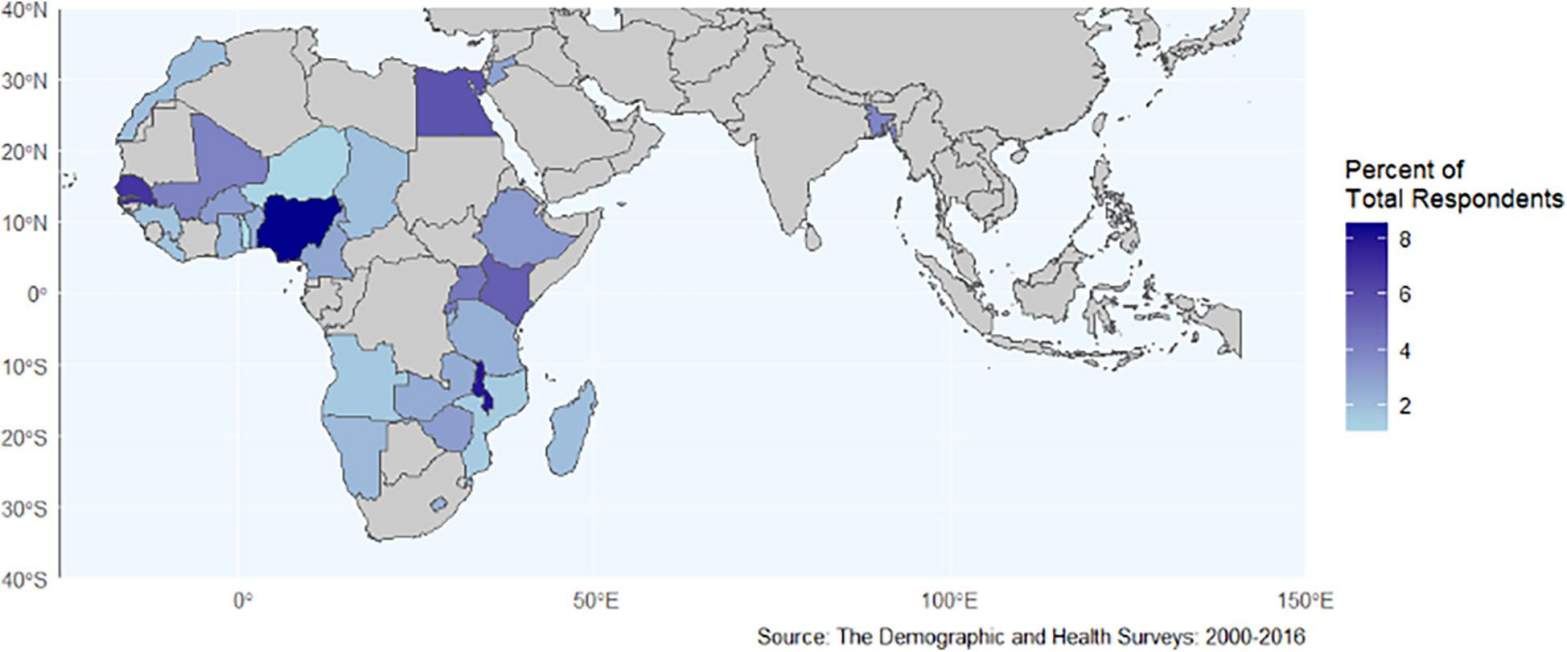
Map of countries included in the study.

To reduce inconsistencies between earlier iterations of DHS survey instruments, we restricted our database to surveys conducted in 2000 or later. Surveys without GPS data were excluded. Only respondents who were women of reproductive age (15–49 years old) who were not pregnant at the time of survey administration and were residents of the surveyed household were eligible for inclusion. Women who were pregnant were excluded because they are asked a different set of questions regarding fertility preferences in the DHS and because DHS surveys automatically code all women who are pregnant as not using contraception. This yielded a sample of 74 surveys from 33 countries (see Figure 1) conducted between 2000 and 2016, with 820,746 individual survey response records.

Twenty-four of the 33 countries in our sample had data from multiple survey years. We included multiple DHS survey waves within countries for several reasons: to ensure adequate statistical power given the rarity of both climate anomaly exposures and certain low-prevalence demographic sub-groups and SRH attitudes and practices; to capture stable, long-term patterns rather than isolated events; to strengthen our fixed effects methodology by leveraging within-country temporal variation; and to maintain consistency with recent comparable research (38).

### 2.3 Sexual and reproductive health attitudes and practices of interest

Our analysis focused on three SRH domains of interest: contraceptive use, fertility preference, and contraceptive autonomy. These selected domains and corresponding IPUMS-harmonized DHS variables were selected for their broad availability and the level of sub-analyses they permitted. For brevity, we provide more detail about the operationalization of our predictor set, including how variables were coded, in Supplementary Material 1: Recoding Strategy.

Within the contraceptive use domain, we considered whether respondents; (1) reported using modern contraception methods at the time of survey administration, (2) reported using traditional contraception, or (3) reported non-use of contraception. Among those using modern contraception, we also examined whether respondents (4) reported using short-acting contraceptive methods, or (5) reported using long-acting contraceptives.

Within the fertility preference domain, we considered whether respondents (6) reported wanting to have a child generally, (7) reported wanting to have a child within the next 2 years, (8) reported wanting to have a child in 2 or more years, and (9) reported that they were certain in their fertility preference.

Within the contraceptive autonomy domain, we considered, of respondents who reported current use of contraception, whether respondents (10) reported that they were the sole decider for contraceptive decision-making or, (11) their husbands were primarily responsible for contraception decision-making. We defined autonomy as cases where women indicated they were exclusively responsible for contraceptive decision-making.

### 2.4 Climate anomalies

We defined climate anomalies as the number of days with localized extreme climatic values in the year before survey administration, with a threshold set as the 95th percentile of measurements over the 1981–2010 baseline reference period (39–42). That is, temperature anomalies (hereafter, “extreme heat”) were defined as the number of days for each survey's cluster-level GPS coordinates where the daily temperature exceeded the 95th percentile of the baseline cluster historical mean, and precipitation anomalies (hereafter, “extreme precipitation”) were defined as the number of days for each survey's cluster-level GPS coordinates where the daily precipitation value exceeded the 95th percentile of the baseline cluster historical mean. We then *z*-scored the anomaly count at the cluster-level to standardize interpretation across climatic measures.

Table 1 shows mean extreme temperature and extreme precipitation days within the 365 days before survey administration across the pooled sample. On balance, precipitation anomalies were rarer events than temperature anomalies. 99.8% (*n* = 819,135) of respondents resided in DHS cluster locations that were exposed to at least one extreme temperature and one extreme precipitation day in the year preceding survey administration.

**Table 1:** Descriptive statistics of exposure to climatic anomalies for the pooled sample.

Anomaly (time horizon)	Mean	Median	Std. Dev.
Extreme heat (365 Days)	24.2	23	10.76
Extreme precipitation (365 days)	18.20	18	5.48

### 2.5 Analytic approach

We adopted an analytic approach similar to that deployed by Gray and Thiede (38) to evaluate the association between the occurrence of climate anomalies (specifically, extreme temperature and extreme precipitation) in the 365 days prior to survey administration and reported contraception use, fertility preferences, and contraceptive autonomy. Specifically, we used fixed-effect logistic regression models that included (a) temperature and precipitation anomalies, (b) cluster-level temperature and precipitation values over the 365-day period before survey completion, (c) socio-demographic control variables, (d) fixed effects for country, (e) fixed effects for year, (f) a region-specific set of fixed effects for the survey month of the year interacted with regions defined as Sub-Saharan Africa, North Africa/Europe, and South/Southeast Asia, (g) DHS survey weights, and (h) corrections for clustering at the level of the DHSID survey cluster via cluster robust standard errors. Our base model can be represented as:

$$\ln\left(\frac{\;p}{1-p}\right)=\alpha_{p}\;+\;a_{r}m\;+\;a_{r}t\;+\;C_{ct}\;+\;BX_{ic},$$

where the log-odds of a value of 1 for woman for an SRH outcome variable is a function of country fixed effects (*α*_p_), region-specific month-of-year effects (*α_r_*m), standardized climate anomalies (C*_ct_*) that are specific to survey cluster *c* and time *t*, and individual and cluster characteristics (*X*). Extensions of this model include a series of models—presented in SM 3: Robustness Checks—which include squared terms of standardized climate anomalies (C*_ct_*) to account for potential nonlinearities in the relationship between climate anomalies and SRH outcome variables of interest as well as interactions between the standardized climate anomaly *z*-scores and baseline climate values in the year before survey administration. We also conducted descriptive analyses to examine exposure to extreme heat by contraceptive method, using data for all known heat-sensitive modern contraceptive methods available in IPUMS-DHS. Analyses were conducted in R. Results from our primary model set (i.e., the odds ratios) can be interpreted as the linear population-level average change in the odds of a given SRH outcome variable associated with a one standard deviation increase in anomaly exposure (e.g., for a 365 day exposure period, the change in the odds of SRH outcome variables associated with being exposed to 34.96 extreme heat days compared to 24.2 extreme heat days compared to 24.2 extreme heat days and the change in odds of SRH outcome variables of interest associated with being exposed to 23.68 extreme precipitation days compared to 18.20 extreme precipitation days).

## 3 Results

### 3.1 Descriptive analyses

#### 3.1.1 Study population

Our study population consisted of 820,746 non-pregnant women of reproductive age from 33 low- and middle-income countries. The mean age of respondents was 29.3 years. Full descriptive statistics are available in Tables 2, 3; the number of observations by country is available in Supplementary Material 2: Respondents by Country.

**Table 2:** Unweighted descriptive statistics for the outcomes and predictors of the pooled sample*.*

Category	Variable	Distribution (n, %)				
		No		Yes		
Contraception use	Any contraception use	580,812 (70.8%)		239,934 (29.2%)		
	Modern contraception use	614,581 (74.9%)		206,165 (25.1%)		
	Traditional contraception use	786,977 (95.9%)		33,769 (4.1%)		
Duration of modern methods	Short-acting contraception use	736,558 (89.7%)		84,188 (10.3%)		
	Long-acting contraception use	700,129 (85.3%)		120,617 (14.7%)		
Method type	Oral contraception pill use	770,799 (93.9%)		49,947 (6.1%)		
	Intrauterine device use	796,080 (97.0%)		24,666 (3.0%)		
	Implant use	805,777 (98.2%)		14,969 (1.8%)		
	Condom use	790,568 (96.3%)		30,178 (3.7%)		
	Injectable contraception use	754,002 (91.9%)		66,744 (8.1%)		
	Female sterilization	807,045 (98.3%)		13,701 (1.7%)		
	Engaged in period of abstinence	802,908 (97.8%)		17,838 (2.2%)		
Fertility preference	Desire for children	348,961 (42.5%)		471,785 (57.5%)		
	Desire for children in the next 2 years	684,112 (83.4%)		136,634 (16.6%)		
	Desire for children in 2 or more years	599,898 (73.1%)		220,848 (26.9%)		
Contraceptive autonomy: among current contraception users	Respondent is sole decision-maker	202,215 (84.3%)		37,719 (15.7%)		
	Husband is sole decision-maker	223,710 (98.0%)		16,224 (2.0%)		
Demographic characteristics	Urban status	Rural		Urban		
		523,769 (63.8%)		296,977 (36.2%)		
	Marital status	Not Married		Married		
		336,310 (41.0%)		484,436 (59.0%)		
	Education level	No Education	Primary	Secondary		Higher
		271,124 (33.0%)	270,731 (33.0%)	239,083 (29.1%)		39,808 (4.9%)
	Wealth quintile	Poorest	Poorer	Middle	Richer	Richest
		156,813 (19.1%)	155,330 (18.9%)	160,338 (19.5%)	163,641 (19.9%)	184,624 (22.5%)
	Husband years of education (quartile)	Missing	Q1	Q2	Q3	Q4
		240,315 (29.3%)	145,108 (17.7%)	145,108 (17.7%)	145,108 (17.7%)	145,108 (17.7%)

**Table 3:** Unweighted descriptive statistics for the outcomes and predictors of the pooled sample (numeric).

Variable	Mean ± SD
Age	29.3 ± 9.7
Number of children	3.0 ± 2.8
Number of household residents	6.8 ± 4.4
Wealth index	0.1 ± 2.4

Of the women in our sample, 29.2% (*n* = 239,934) reported use of any contraceptive method, with 25.1% (*n* = 206,178) of our sample using modern contraception methods and 4.1% (*n* = 33,769) using traditional contraception methods. 70.8% (*n* = 580,812) of respondents reported not using any contraceptive method. By type of modern contraceptive method used, 10.3% (*n* = 84,194) of our sample reported use of short-acting contraceptive methods and 14.7% (*n* = 120,624) reported use of long-acting methods. By modern contraceptive method, 6.1% (*n* = 49,947) of our sample reported using oral contraceptive pills, 3.0% (*n* = 24,666) used intrauterine devices (IUDs), 1.8% (*n* = 14,969) used implants, 3.7% (*n* = 30,178) used condoms, 8.1% (*n* = 66,744) used injectable contraception, and 1.7% (*n* = 13,701) reported using sterilization. Regarding use of traditional contraceptive methods, 2.2% (*n* = 17,383) of our sample reported engaging in periods of abstinence as their contraceptive method of choice.

Within our sample, 57.5% (*n* = 471,819) of women respondents reported wanting to have a child generally, with 16.6% (*n* = 136,634) reporting wanting to have a child within the next 2 years and 26.9% (*n* = 220,848) reporting wanting to have a child in 2 or more years. 72.7% of respondents (*n* = 59,675,911) reported that they were certain in their fertility preferences.

Finally, of respondents who reported current use of contraception, 15.7% (*n* = 37,719) reported that they were the sole decider for contraceptive decision-making, 6.8% (*n* = 16,224) reported that their husbands were primarily responsible for contraception decision-making, and 57.4% reported joint-decision-making (*n* = 137,868).

#### 3.1.2 Exposure to extreme heat by modern contraceptive method type

In addition to the impact of climate change on SRH behaviors and preferences, we also considered other potential impacts, including the potential exposure of a range of contraceptive products to temperatures exceeding WHO's storage guidelines. We considered heat-sensitive contraception methods as those (1) included within the DHS dataset and (2) for which WHO has provided temperature storage and exposure recommendations; these methods include condoms, implants, oral contraception pills, injectable contraception, and IUDs). Table 4 presents the percentage of sampled respondents residing in areas where the 365-day rolling mean temperature exceeded the standard 30°C shelf-stability temperature recommendations for heat-sensitive contraceptive methods (29, 30). Notably, 55.2% (*n* = 453,071) of the pooled sample respondents lived in regions where the rolling mean temperature in the year preceding survey administration surpassed the shelf-stability threshold of 30°C and, of the subset who reported using heat-sensitive contraception methods, 41.4% (*n* = 186,504) of respondents lived in regions where the rolling mean temperature in the year preceding the survey surpassed the storage stability threshold of 30°C. Specifically, 49.4% of condom users (*n* = 14,917), 47.4% of implant users (*n* = 7,088), 46.4% of oral contraceptive pill users (*n* = 23,178), 40.6% of injectable contraception users (*n* = 27,075), and 20.1% of IUD users (*n* = 4,959) lived in these heat-exposed regions.

**Table 4:** How the use of heat-sensitive contraception varies by rolling mean temperatures.

Contraception method	Mean temperature >30°C
Injectables (*n* = 66,744)	40.6% (*n* = 27,075)
Pill (*n* = 49,947)	46.4% (*n* = 23,178)
Condoms (*n* = 30,178)	49.4% (*n* = 14,917)
IUD (*n* = 24,666)	20.10% (*n* = 4,959)
Implants (*n* = 14,969)	47.4% (*n* = 7,088)

### 3.2 Pooled sample results

In this section, we report the results of analyses evaluating the association of climate anomalies with a suite of outcome variables focused on specific SRH attitudes and practices for the pooled sample (i.e., across all groups and countries). Generally, we evaluated the association of exposure to extreme heat and extreme precipitation with contraception use, fertility preferences, and contraceptive autonomy across a series of 22 models. Our linear model results represent our primary findings, supported by country-level robustness checks (see Supplementary Material 3: Robustness Checks).

#### 3.2.1 Pooled sample results: contraception use

To assess the association between exposure to climate anomalies and contraception use, we explored how anomaly exposure was associated with use of contraception generally, including (1) use of modern contraception, (2) use of traditional contraception, and (3) the non-use of contraception. We also examined specific trends among modern contraception users, namely (4) the use of short-acting contraception and (5) the use of long-acting contraception.

In Table 5, we report the odds ratios for the association between exposure to extreme heat and precipitation and use of modern contraception, traditional contraception, and non-use of contraception. In Table 6, we report the odds ratios for association between extreme heat and precipitation exposure and the use of short-acting contraception and long-acting contraception.

**Table 5:** Association of 365-day temperature and precipitation anomalies with modern and traditional contraception use and non-use of any contraception (*n* = 820,746)*.*

Predictor variable	Dependent variable					
	Modern contraception use		Traditional contraception use		No contraception use	
	(1)	(2)	(3)	(4)	(5)	(6)
Temperature anomaly	0.97***	–	0.96***	–	1.03***	–
Temperature mean (365 days)	0.95***	–	0.92***	–	1.06***	–
Precipitation anomaly	–	1.00***	–	1.01***	–	1.01
Precipitation sum (365 days)	–	1.00***	–	1.00***	–	1.00***
Age	1.37***	1.37***	1.26***	1.25***	0.74***	0.74***
Age squared	0.99***	0.99***	1.00***	1.00***	1.01***	1.01**
Urban-rural status: urban	1.26***	1.27***	1.10***	1.11***	0.81***	0.81***
Marital status: married	1.95***	1.96***	1.90***	1.94***	0.51***	0.51***
Education level: higher	2.21***	2.23***	3.51***	3.46***	0.41***	0.41***
Education level: primary	1.64***	1.64***	1.67***	1.64***	0.61***	0.50***
Education level: secondary	2.11***	2.11***	2.58***	2.52***	0.46***	0.46***
Number of children birthed	1.17***	1.16***	1.12***	1.11***	0.87***	0.87***
Count of household residents	0.99***	0.99***	0.99***	0.99***	1.01***	1.01***
Wealth quintile: middle	0.76***	0.77***	0.78***	0.79***	1.30***	1.29***
Wealth quintile: poorer	0.69***	0.69***	0.69***	0.70***	1.45***	1.45***
Wealth quintile: poorest	0.56***	0.56***	0.61***	0.61***	1.75***	1.75***
Wealth quintile: richer	0.86***	0.87***	0.84***	0.85***	1.17***	1.15***
Household wealth index	0.99***	1.00	1.00***	0.99*	1.01***	1.00***
Husband education years: missing	1.00	1.00	1.00***	1.10***	0.98***	0.98***
Husband education years: second quartile	1.47***	1.47***	1.48***	1.45***	0.68***	0.68***
Husband education years: third quartile	1.47***	1.46***	1.63***	1.60***	0.67***	0.68***
Husband education years: fourth quartile	1.48***	1.46***	1.82***	1.77***	0.65***	0.66***
Observations	820,746	820,746	820,746	820,746	820,746	820,746

Modern contraception use and traditional contraception use models are multinomial logistic regressions with reference group “No Contraception Use” (0). No contraception use models are binary logistic regressions with reference category “Contraception Use”. Constant, country, region, month of the year, and year-fixed effects are also included in the model. Standard errors are clustered at the level of the DHS survey cluster. Reference categories for categorical variables represent the most frequent response. The reference category for Marital Status is Unmarried. The reference category for Urban is Rural. The reference category for education level is none. The reference category for wealth is richest. The reference category for husband education years is the lowest quartile of education years.

* *p* < .05.

** *p* < .01.

*** *p* < .001.

**Table 6:** Association of 365-day temperature and precipitation anomalies with use of short-acting contraception and long-acting contraception (*n* = 820,746).

Predictor variable	Dependent variable:			
	Short-acting contraception		Long-acting contraception	
	(7)	(8)	(9)	(10)
Temperature anomaly	0.96***	–	0.98***	–
Temperature mean (365 days)	0.96***	–	0.93***	–
Precipitation anomaly	–	0.97***	–	1.03***
Precipitation sum (365 days)	–	1.00***	–	1.00***
Age	1.38***	1.38***	1.39***	1.39***
Age squared	0.99***	0.99***	0.99***	0.99***
Urban-rural status: urban	1.30***	1.31***	1.22***	1.24***
Marital status: married	1.84***	1.85***	2.17***	2.17***
Education level: higher	2.86***	2.87***	1.60***	1.63***
Education level: primary	1.68***	1.68***	1.61***	1.64***
Education level: secondary	2.47***	2.46***	1.76***	1.79***
Number of children birthed	1.09***	1.09***	1.21***	1.21***
Count of household residents	1.09***	0.98***	0.99***	0.99***
Wealth quintile: middle	0.71***	0.72***	0.82***	0.83***
Wealth quintile: poorer	0.62***	0.63***	0.75***	0.75***
Wealth quintile: poorest	0.52***	0.52***	0.61***	0.60***
Wealth quintile: richer	0.81***	0.82***	0.92***	0.92***
Household wealth index	0.98***	0.99***	1.00	1.00**
Husband education years: missing	1.30***	1.29***	0.74***	0.74***
Husband education years: second quartile	1.50***	1.49***	1.38***	1.38***
Husband education years: third quartile	1.55***	1.54***	1.38***	1.38***
Husband education years: fourth quartile	1.63***	1.45***	1.61***	1.43***
Observations	820,746	820,746	820,746	820,746

Short-acting and long-acting contraception models are multinomial logistic regressions with reference group “No Contraception Use” (0). Constant, country, region, month of the year, and year-fixed effects are also included in the model. Standard errors are clustered at the level of the DHS survey cluster. Reference categories for categorical variables represent the most frequent response. The reference category for marital status is unmarried. The reference category for Urban is Rural. The reference category for education level is none. The reference category for wealth is richest. The reference category for husband education years is the lowest quartile of education years.

*p < .05.

** *p* < .01.

*** *p* < .001.

We found modest associations between exposure to extreme heat and precipitation in the 365 days prior to survey administration and patterns of contraceptive use, with exposure to extreme heat and precipitation both associated with lower odds of modern contraception use and higher odds of non-use of contraception (Table 5). For every standard deviation increase in exposure to extreme heat, the odds of reporting modern contraception use were 3% lower (Model 1; OR = 0.97; *P* < 0.001), the odds of reporting traditional contraception use were 4% lower (Model 3; OR = 0.96; *P* < 0.001), and the odds of reporting not using any contraception method were 3% higher (Model 5; OR = 1.03; *P* < 0.001). For every standard deviation increase in exposure to extreme precipitation, the odds of reporting modern contraception use were not substantively different (Model 2; OR = 1.00; *P* < 0.001), the odds of reporting traditional contraception use were 1% higher (Model 4; OR = 1.01; *P* < 0.001), and the odds of reporting not using any contraception method were 1% higher (OR = 1.01; *P* > 0.05), though this finding was not significant at conventional thresholds.

Similarly, we found modest associations between exposure to extreme heat and precipitation in the 365 days prior to survey administration and different types of modern contraception use, with exposure to both extreme heat and precipitation associated with lower odds of short-acting contraception use (Table 6). For every standard deviation increase in exposure to extreme heat, the odds of reporting short-acting contraception use were 4% lower (Model 7; OR = 0.96; *P* < 0.001) and the odds of reporting long-acting contraception use were 2% lower (Model 9; OR = 0.98; *P* < 0.001). For every standard deviation increase in exposure to extreme precipitation, the odds of reporting short-acting contraception use were 3% lower (Model 8; OR = 0.97; *P* < 0.001) and the odds of reporting long-acting contraception use were 3% higher (Model 10; OR = 1.03; *P* < 0.001).

#### 3.2.2 Pooled sample results: fertility preferences

To evaluate the association between exposure to climate anomalies and fertility preferences, we explored how anomaly exposure was associated with (1) the desire to have a child, (2) the desire to have a child within the next 2 years, (3) the desire to have a child in 2 or more years, and (4) the decisiveness of one's fertility preferences.

In Table 7, we report the odds ratios for the association between exposure to extreme heat and precipitation and the desire to have a child, the desire to have a child within the next 2 years, and the desire to have a child in 2 or more years. In Table 8, we report the odds ratios for the associations between exposure to extreme heat and precipitation and the decisiveness of a respondent's fertility preferences.

**Table 7:** Association of 365-day temperature and precipitation anomalies with desire to have children and desire to have children within the next 2 years or more than 2 years (*n* = 820,746).

Predictor variable	Dependent variable:					
	Desire for children		Desire for children (in next 2 years)		Desire for children (in 2 + years)	
	(11)	(12)	(13)	(14)	(15)	(16)
Temperature anomaly	0.96***	–	1.02***	–	0.97***	–
Temperature mean (365 days)	1.05***	–	1.06***	–	0.99***	–
Precipitation anomaly	–	0.99***	–	1.00	–	0.98***
Precipitation sum (365 days)	–	1.00***	–	1.00***	–	1.00***
Age	1.12***	1.12***	1.40***	1.40***	1.27***	1.26***
Age squared	1.00***	1.00***	1.00***	1.00***	1.00***	1.00***
Urban-rural status: urban	0.89***	0.89***	0.92***	0.92***	0.94***	0.94***
Marital status: married	1.95***	1.94***	2.75***	2.75***	1.84***	1.84***
Education level: higher	1.29***	1.28***	0.72***	0.72***	1.82***	1.83***
Education level: primary	0.85***	0.85***	0.81***	0.80***	1.02***	1.02***
Education level: secondary	0.97***	0.96***	0.65***	0.64***	1.34***	1.34***
Number of children birthed	0.69***	0.69***	0.63***	0.63***	1.15***	1.15***
Count of household residents	0.99***	0.99***	0.97***	0.97***	1.00***	1.00***
Wealth quintile: middle	1.05***	1.05***	1.08***	1.07***	0.93***	0.93***
Wealth quintile: poorer	1.10***	1.10***	1.12***	1.11***	0.93***	0.93***
Wealth quintile: poorest	1.13***	1.14***	1.14***	1.14***	0.91***	0.91***
Wealth quintile: richer	1.05***	1.05***	1.05***	1.04***	0.96***	0.96***
Household wealth index	1.00**	1.00**	1.00***	1.00***	0.99***	0.99***
Husband education years: missing	0.47***	0.47***	0.32***	0.31***	0.52***	0.52***
Husband education years: Second quartile	0.94***	0.94***	0.93***	0.92***	0.93***	0.93***
Husband education years: third quartile	0.79***	0.79***	0.87***	0.86***	0.86***	0.86***
Husband education years: fourth quartile	0.73***	0.74***	0.87***	0.86***	0.88***	0.88***
Observations	820,746	820,746	820,746	820,746	820,746	820,746

Fertility preferences models are binary logistic regressions. For Models 11 and 12, the reference category is “No Desire to Have Children” (0). For Models 13 and 14, the reference category is “Does Not Desire to Have Children in the Next Two Years” (0). For Models 15 and 16, the reference category is “Does Not Desire to Have Children in Two or More Years” (0). Constant, country, region, month of the year, and year-fixed effects are also included in the model. Standard errors are clustered at the level of the DHS survey cluster. Reference categories for categorical variables represent the most frequent response. The reference category for marital status is unmarried. The reference category for urban is rural. The reference category for education level is none. The reference category for wealth is richest. The reference category for husband education years is the lowest quartile of education years.

*p < .05.

** *p* < .01.

*** *p* < .001.

**Table 8:** Association of 365-day temperature and precipitation anomalies with decisiveness of fertility preferences (*n* = 820,746).

Predictor variable	Dependent variable:	
	Fertility decisiveness	
	(17)	(18)
Temperature anomaly	0.97***	–
Temperature mean (365 days)	0.98***	–
Precipitation anomaly	–	1.01***
Precipitation sum (365 days)	–	1.00***
Age	1.18***	1.18***
Age squared	1.00***	1.00**
Urban-rural status: urban	1.01***	1.01***
Marital status: married	3.08***	3.08***
Education Level: Higher	1.28***	1.28***
Education level: primary	1.03***	1.03***
Education level: secondary	1.11***	1.12***
Number of children birthed	1.08***	1.08***
Count of household residents	0.98***	0.98***
Wealth quintile: middle	0.95***	0.96***
Wealth quintile: poorer	0.96***	0.97***
Wealth quintile: poorest	0.95***	0.95***
Wealth quintile: richer	0.96***	0.96***
Household wealth index	0.98***	0.98***
Husband education years: missing	0.24***	0.25***
Husband education years: second quartile	1.11***	1.11***
Husband education years: third quartile	1.07***	1.08***
Husband education years: fourth quartile	1.12***	1.12***
Observations	820,746	820,746

Decisive fertility preferences models are binary logistic regressions with reference category “Not Decisive” (0 = indecisive fertility preferences). Constant, country, region, month of the year, and year-fixed effects are also included in the model. Standard errors are clustered at the level of the DHS survey cluster. Reference categories for categorical variables represent the most frequent response. The reference category for marital status is unmarried. The reference category for urban is rural. The reference category for education level is none. The reference category for wealth is richest. The reference category for husband education years is the lowest quartile of education years.

*p < .05.

** *p* < .01.

*** *p* < .001.

We found modest associations between exposure to extreme heat and precipitation in the 365 days prior to survey administration and fertility preferences, with exposure to both extreme heat and precipitation associated with lower odds of reporting desire for children generally and in 2 or more years (Table 7). For every standard deviation increase in exposure to extreme heat, the odds of reporting a desire to have children were 4% lower (Model 11; OR = 0.96; *P* < 0.001), the odds of desiring to have children within the next 2 years were 2% higher (Model 13; OR = 1.020; *P* < 0.001), and the odds of desiring to have children in 2 or more years were 3% lower (Model 15; OR = 0.970; *P* < 0.001). For every standard deviation exposure to extreme precipitation, the odds of reporting a desire to have children were 1% lower (Model 12; OR = 0.99; *P* < 0.001), the odds of desiring to have children within the next 2 years were not substantively different (Model 14; OR = 1.00; *P* < 0.001), and the odds of desiring to have children in 2 or more years were 2% lower (Model 16; OR = 0.98; *P* < 0.001).

Associations between exposure to extreme heat and precipitation in the 365 days prior to survey administration and decisiveness of fertility preferences were modest (Table 8). For every standard deviation increase in exposure to extreme heat, the odds of reporting decisive fertility preferences were 3% lower (Model 17; OR = 0.97; *P* < 0.001). For every standard deviation increase in exposure to extreme precipitation, the odds of reporting decisive fertility preferences were 1% higher ((Model 18; OR = 1.01; *P* < 0.001).

#### 3.2.3 Pooled sample results: contraceptive autonomy

To evaluate the association of exposure to climate anomalies with contraceptive autonomy among current contraception users, we explored how anomaly exposure was associated with (1) sole decision-making over contraceptive use by the respondent and (2) joint decision-making with husband, compared to situations where the husband was the primary decision-maker.

In Table 9, we report the odds ratios for the association between exposure to extreme heat and precipitation and whether a respondent reported sole decision-making over contraceptive use and joint decision-making with their husband.

**Table 9:** Association of 365-day temperature and precipitation anomalies with contraceptive autonomy (*n* = 191,816).

Predictor variable	Dependent variable			
	Solo decision-making		Joint-decision making	
	(19)	(20)	(21)	(22)
Temperature anomaly	1.06***	–	1.03***	–
Temperature mean (365 days)	0.99***	–	0.98***	–
Precipitation anomaly	–	1.01***	–	1.06***
Precipitation sum (365 days)	–	1.00***	–	1.00***
Age	1.06***	1.06***	1.04***	1.04***
Age squared	1.00***	1.00***	1.00***	1.00***
Urban-rural status: urban	1.16***	1.17***	0.98	0.99
Marital status: married	0.54***	0.55***	0.97***	0.97***
Education level: higher	1.18***	1.18***	1.44***	1.44***
Education level: primary	1.08***	1.08***	1.10***	1.10***
Education level: secondary	1.23***	1.23***	1.29***	1.29***
Number of children birthed	1.02***	1.02***	1.00	1.00
Count of household residents	1.00	1.00	1.00	1.00
Wealth quintile: middle	0.99	0.99	0.95***	0.95***
Wealth quintile: poorer	1.12***	1.11***	1.01	1.01
Wealth quintile: poorest	1.15***	1.14***	0.93***	0.92***
Wealth quintile: richer	1.03	1.03	0.99	0.99
Household wealth index	1.01	1.01	1.02***	1.02***
Husband education years: missing	1.18***	1.18***	1.04***	1.04***
Husband education years: second quartile	0.84***	0.85***	1.08***	1.08***
Husband education years: third quartile	0.76***	0.76***	1.09***	1.08***
Husband education years: fourth quartile	0.73***	0.72***	1.08***	1.08***
Observations	191,816	191,816	191,816	191,816

Reproductive autonomy models are multinomial logit models with reference group “Husband Decides.” Constant, country, region, month of the year, and year-fixed effects are also included in the model. Standard errors are clustered at the level of the DHS survey cluster. Reference categories for categorical variables represent the most frequent response. The reference category for Marital Status is Unmarried. The reference category for Urban is Rural. The reference category for Education Level is None. The reference category for Wealth is Richest. The reference category for Husband Education Years is the lowest quartile of education years.

*p < .05.

**p < .01.

*** *p* < .001.

We observed modest associations between exposure to extreme heat and precipitation in the 365 days before survey administration and contraceptive autonomy, with exposure to extreme heat and precipitation associated with higher odds of women being the sole decider over contraception use (Table 9). For every standard deviation increase in exposure to extreme heat, the odds of reporting sole decision-making over contraceptive use were 6% higher (Model 19; OR = 1.06; *P* < 0.001) and the odds of reporting joint-decision making were 3% higher (Model 21; OR = 1.03; *P* < 0.001). For every standard deviation increase in exposure to extreme precipitation, the odds of reporting sole decision-making over contraceptive use were 1% higher (Model 20; OR = 1.01; *P* < 0.001) and the odds of reporting joint contraceptive decision making were 6% higher (Model 22; OR = 1.06; *P* < 0.001).

### 3.3 Sociodemographic sub-group analysis

**Figure 2:**
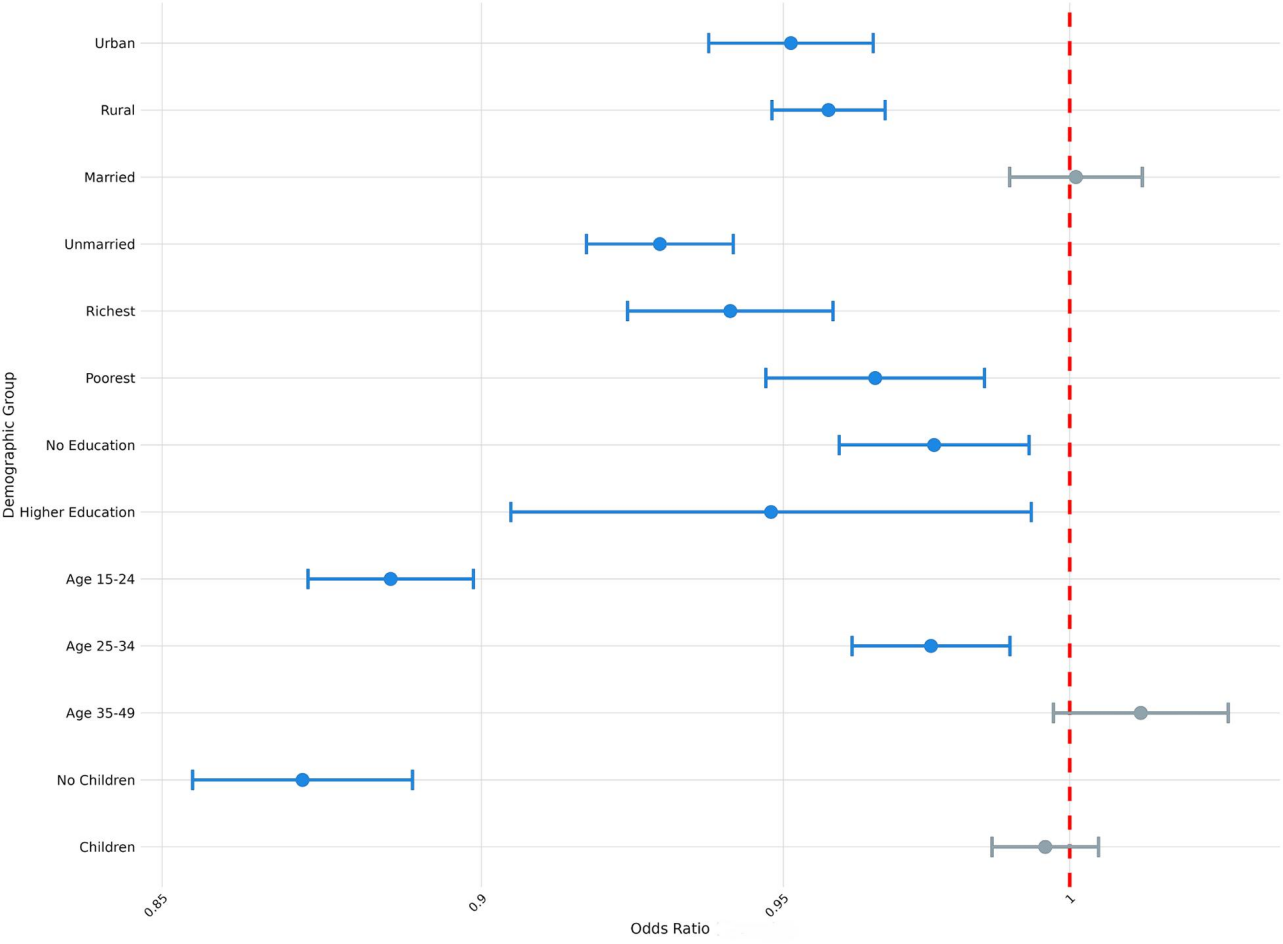
Odds ratio (log-scale) of the association between desire for children and exposure to temperature extremes (survey weighted), by sociodemographic sub-group. Blue denotes statistically significant results and grey denotes non-significant results. 95% confidence intervals; all *p* < 0.05.

**Figure 3:**
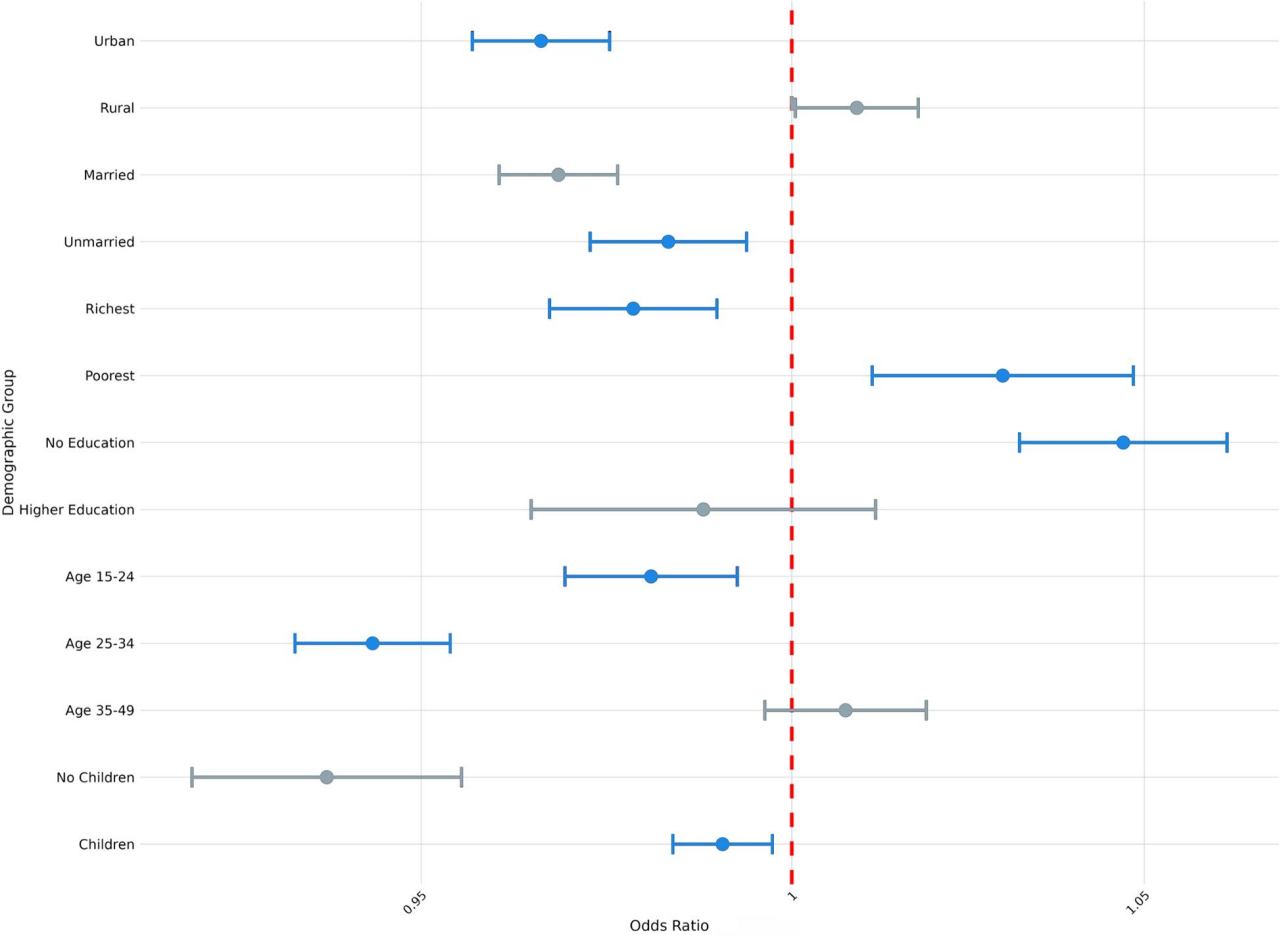
Odds ratio (log-scale) of the association between use of modern contraception and exposure to precipitation extremes (survey weighted), by sociodemographic sub-group. Blue denotes statistically significant results and grey denotes non-significant results. 95% confidence intervals; all *p* < 0.05.

To account for heterogeneity in exposure among sociodemographic groups, we also examined how exposure to climate anomalies was associated with modern contraceptive use, contraceptive autonomy, and fertility preferences across samples that varied by parity, age, education status, household wealth, marital status, and urban-rural status. Here, we highlight two areas of particular interest: the association between exposure to extreme heat and fertility preferences (Figure 2) and the association between exposure to extreme precipitation and modern contraception use (Figure 3), by parity, age, education status, household wealth, marital status, and urban-rural status. We present full results of stratified regressions in Supplementary Material 4: Sociodemographic Sub-Analyses.

From Figure 2, we observe variation in the association between exposure to extreme heat and fertility preferences conditional on respondents' different demographic attributes (e.g., parity, age, marital status, etc.). For example, whereas exposure to extreme heat was not significantly associated with the desire to have children among respondents who reported previously having had children (OR = 1.00; *P* > 0.05), exposure to extreme heat was associated with 13% lower odds of reporting desire to have children among respondents who had not previously had children (OR = 0.87; *P* < 0.001). Similarly, whereas exposure to extreme heat was not significantly associated with the desire to have children among the oldest age cohort (aged 35–49; OR = 1.01; *P* > 0.05), exposure to extreme heat was associated with 11% lower odds of reporting desire to have children among the youngest cohort of respondents (aged 15–24; OR = 0.89; *P* < 0.001).

From Figure 3, we similarly observe variation in the association between exposure to extreme precipitation and the use of modern contraception, conditional on different demographic attributes of respondents. For example, exposure to extreme precipitation was associated with 1% lower odds of using modern contraception among respondents who reported previously having had children (OR = 0.99; *P* < 0.01), compared to 6% lower odds of using modern contraception among respondents without children (OR = 0.94; *P* < 0.001). Similarly, exposure to extreme precipitation was associated with 3% lower odds of modern contraception use among urban respondents (OR = 0.97; *P* < 0.001) and 3% lower odds of modern contraception use among the wealthiest cohort (OR = 0.97; *P* < 0.001), whereas exposure to extreme precipitation was not associated with changed odds of modern contraception use among rural respondents and 3% higher odds of modern contraception use among the poorest cohort (OR = 1.03; *P* < 0.01).

### 3.4 Country sub-group analysis

**Figure 4:**
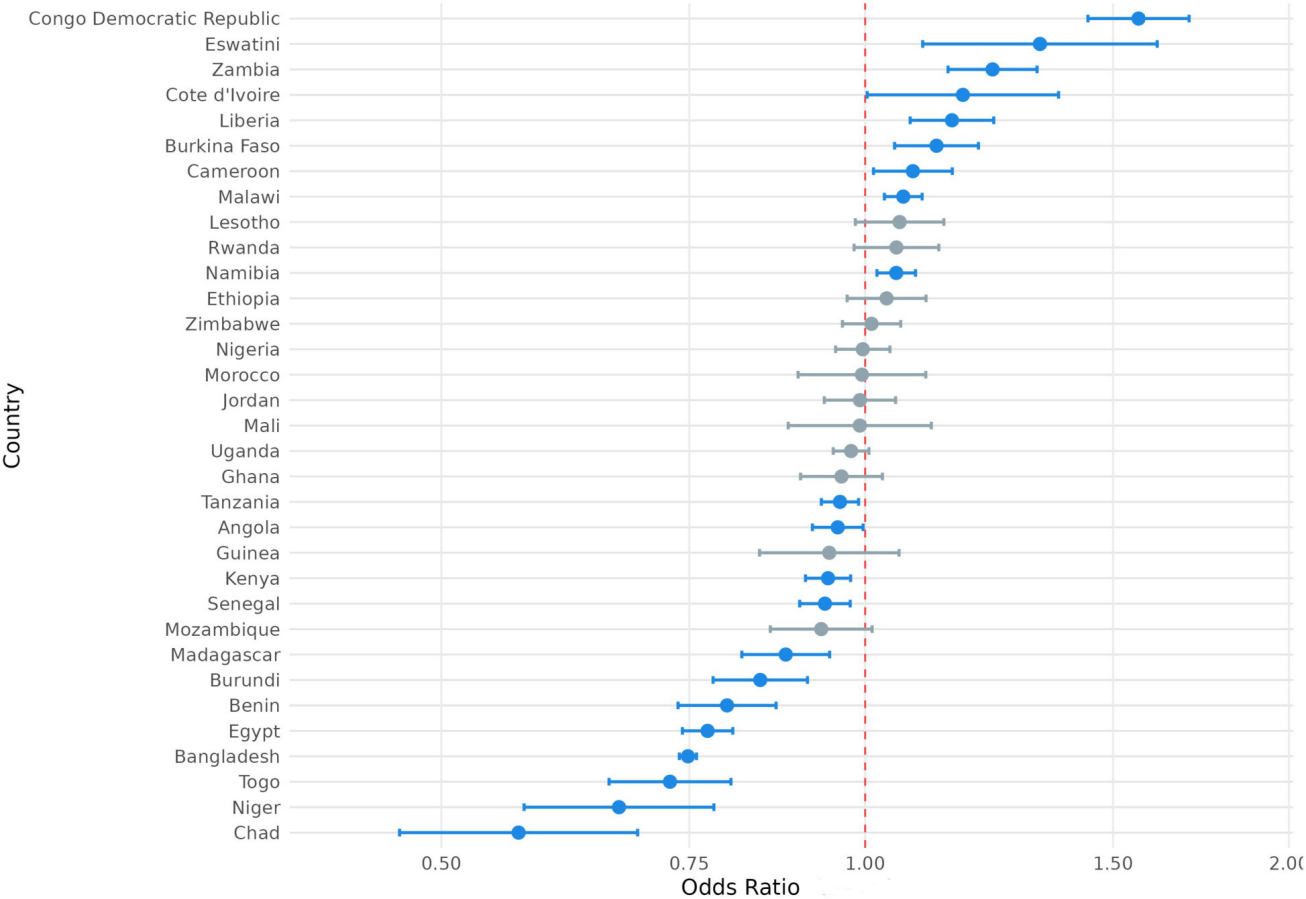
Odds ratio (log-scale) of the association between use of modern contraception and exposure to temperature extremes, by country. Blue denotes statistically significant results and grey denotes non-significant results. 95% confidence intervals; all *p* < 0.05.

**Figure 5:**
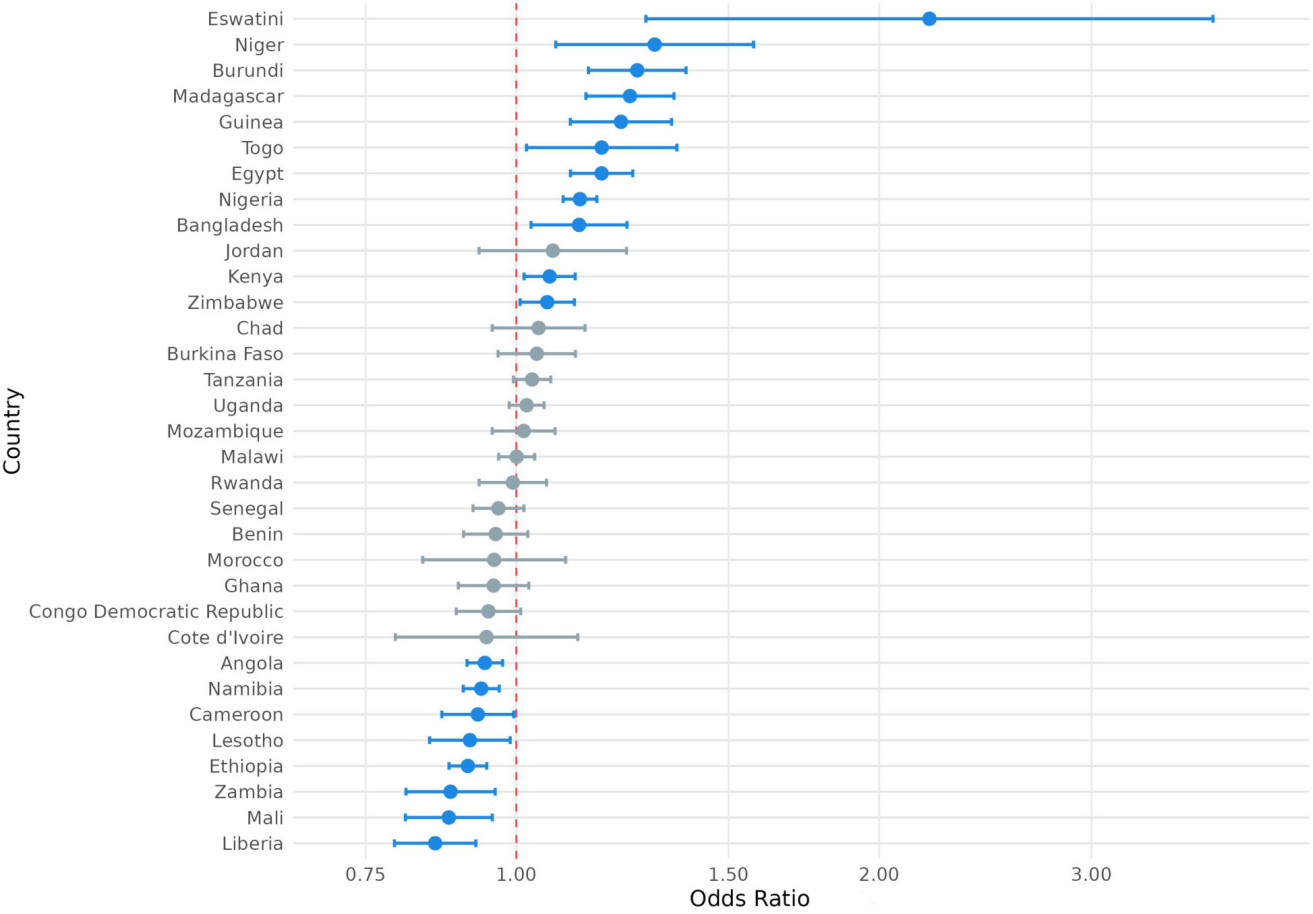
Odds ratio (log-scale) of the association between desire for children associated and exposure to temperature extremes, by country. Blue denotes statistically significant results and grey denotes non-significant results. 95% confidence intervals; all *p* < 0.05.

**Figure 6:**
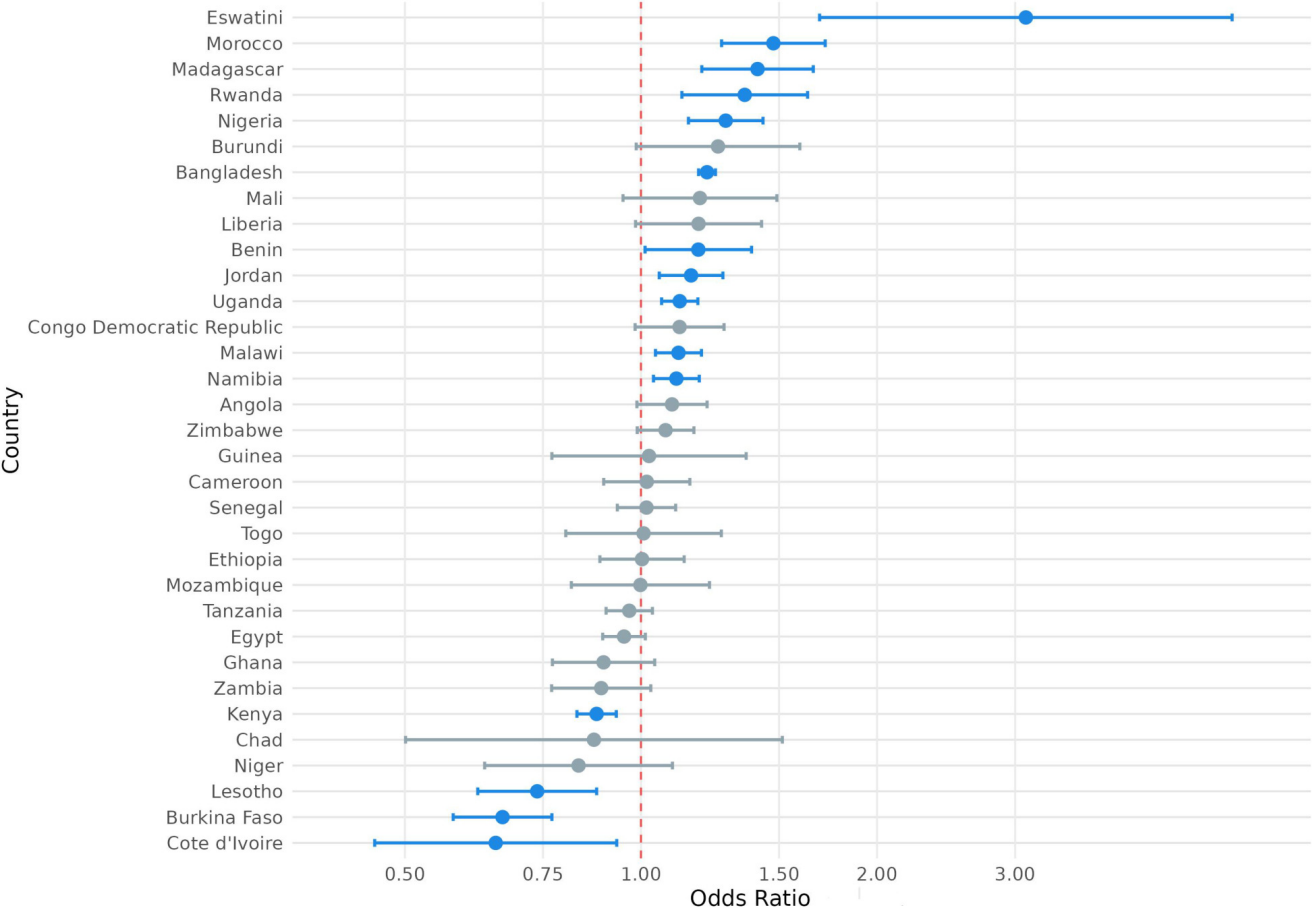
Odds ratio (log-scale) of the association between contraceptive autonomy and exposure to temperature extremes, by country. Blue denotes statistically significant results and grey denotes non-significant results. 95% confidence intervals; all *p* < 0.05.

**Figure 7:**
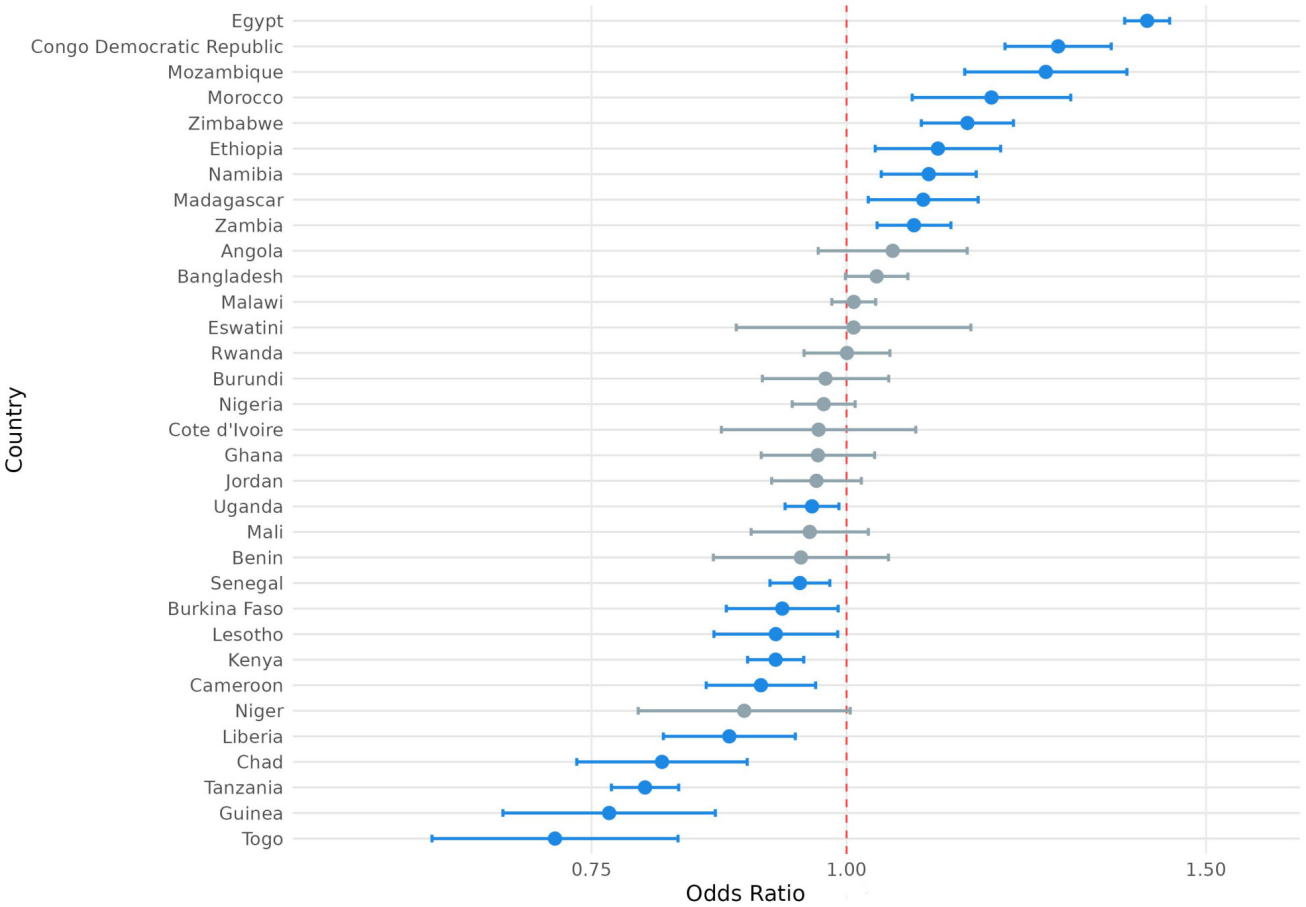
Odds ratio (log-scale) of the association between use of modern contraception and exposure to precipitation extremes, by country. Blue denotes statistically significant results and grey denotes non-significant results. 95% confidence intervals; all *p* < 0.05.

**Figure 8:**
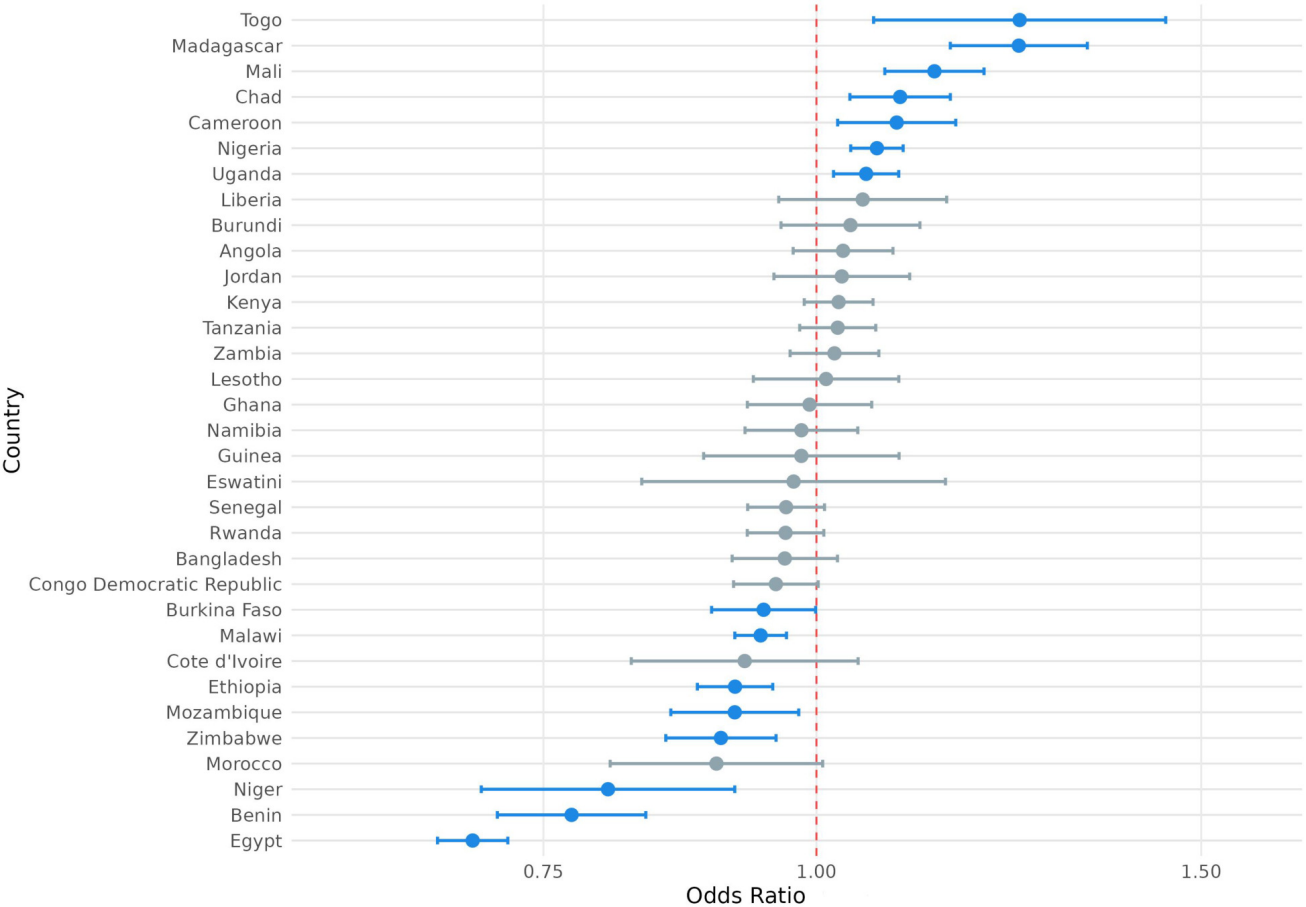
Odds ratio (log-scale) of the association between desire for children and exposure to precipitation extremes, by country. 95% confidence intervals; all *p* < 0.05.

**Figure 9:**
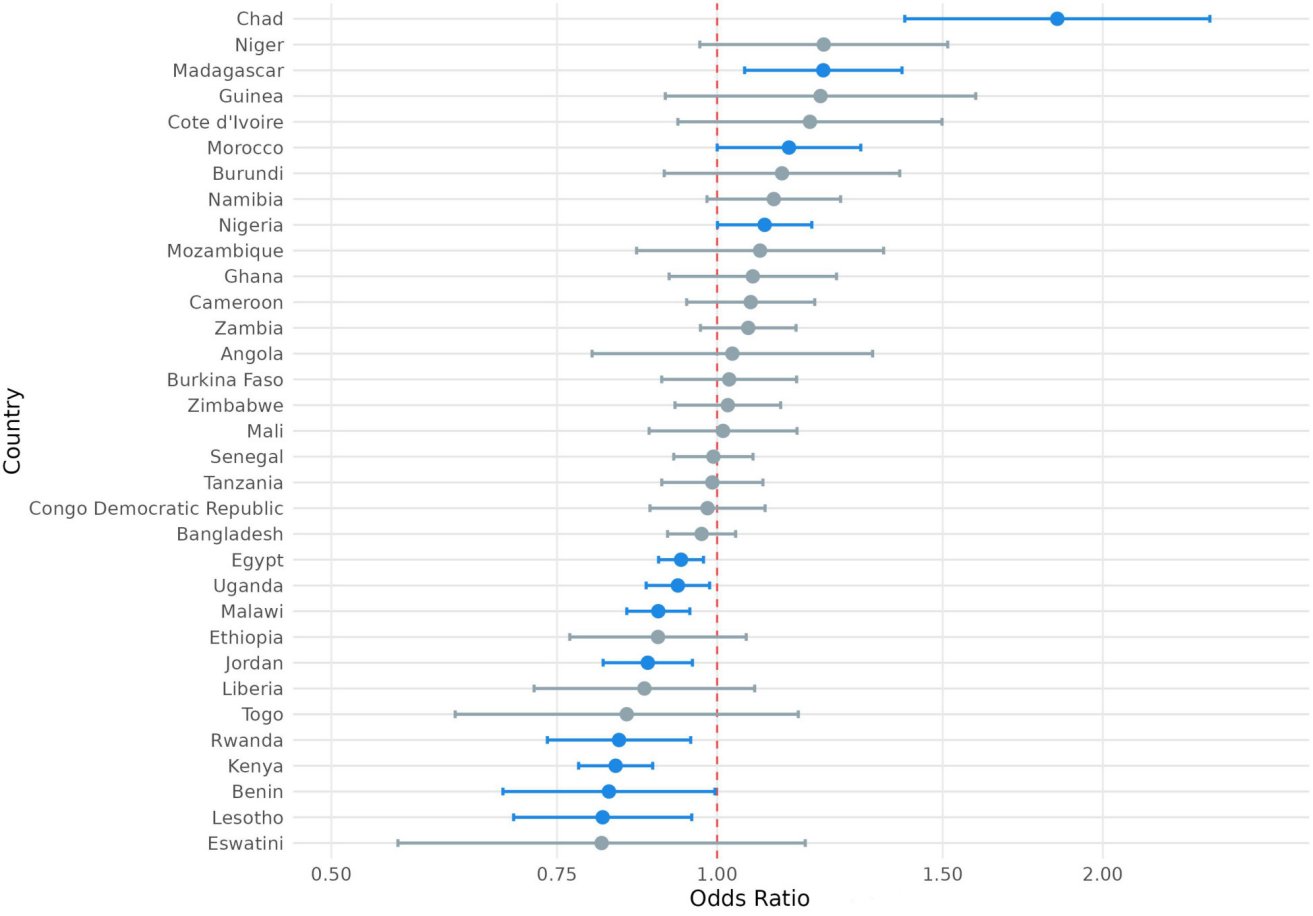
Odds ratio (log-scale) of the association between contraceptive autonomy and exposure to precipitation extremes, by country. Blue denotes statistically significant results and grey denotes non-significant results. 95% confidence intervals; all *p* < 0.05.

We also considered the possibility that exposure to climate anomalies could have heterogenous relationships with the SRH outcome variables of interest among respondents in different countries. To this end, we present the country-level odds ratios for the association between exposure to extreme heat and modern contraception use, contraceptive autonomy, and fertility preference in Figures 4–6 and country-level odds ratios for the association beteen exposure to extreme precipitation and modern contraception use, contraceptive autonomy, and fertility preference in Figures 7–9. We present country-level odds ratio estimates in Supplementary Material 5: Country-Level Analyses for Key SRH Attitudes and Practices. Figures 4–6 suggest that there is considerable heterogeneity across countries in the relationship between extreme heat exposure and the SRH outcome variables of interest. For example, exposure to extreme heat was significantly associated with reported modern contraception use in 21 countries, with a range of 43% lower odds of modern contraception use in Chad (OR = 0.567, *P* < 0.01) to 56% higher odds of modern contraception use in the Democratic Republic of the Congo (OR = 1.56, *P* < 0.01). Exposure to extreme heat was significantly associated with reported desire to have children in 19 countries, with a range of 14% lower odds of reporting desire for children in Liberia (OR = 0.86, *P* < 0.001) to 120% higher odds of reporting desire for children in Eswatini (OR = 2.20, *P* < 0.001). Exposure to extreme heat was significantly associated with reported contraceptive autonomy (respondent as the sole decider on use of contraception) in 15 countries, with a range of 35% lower odds of reporting contraceptive autonomy in Cote d'Ivoire (OR = 0.65, *P* < 0.01) to 210% higher odds of reporting contraceptive autonomy in Eswatini (OR = 3.10, *P* < 0.01).

Similar variation was observed with the association between exposure to extreme precipitation and SRH outcome variables of interest (Figures 7–9). For example, exposure to extreme precipitation was significantly associated with reported modern contraception use in 20 countries, with a range of 28% lower odds of modern contraception use in Togo (OR = 0.72, *P* < 0.001) to 40% higher odds of modern contraception use in Egypt (OR = 1.40, *P* < 0.001). Exposure to extreme precipitation was significantly associated with reported desire for children in 15 countries, with a range of 30% lower odds of reporting desire for children in Egypt (OR = 0.70, *P* < 0.001) to 24% higher odds of reporting desire for children in Togo (OR = 1.24, *P* < 0.001). Exposure to extreme precipitation was significantly associated with reported contraceptive autonomy in 12 countries, with a range of 19% lower odds of reporting contraceptive autonomy in Lesotho (OR = 0.81, *P* < 0.001) to 84% higher odds of reporting contraceptive autonomy in Mozambique (OR = 1.84, *P* < 0.001).

### 3.5 Double exposure and contraception use, contraceptive autonomy, and fertility preference

We also considered whether double exposures (i.e., exposure to both extreme heat and precipitation) were associated with trends in modern contraception use, contraceptive autonomy, and fertility preference. Since 99.8% of our sample experienced at least one extreme heat and one extreme precipitation day in the year prior to survey administration, we created a dummy variable set equal to one in cases where the respondent's location (e.g., their DHS survey cluster) experienced both (1) extreme heat exposure greater than or equal to one standard deviation above the sample mean and (2) extreme precipitation exposure greater than or equal to one standard deviation above the sample mean; this was equivalent to more than 34.96 days of extreme heat exposure and more than 23.68 days of extreme precipitation exposure.

Under this operationalization, double exposures were relatively uncommon in our sample, occurring for only 2.7% of respondents (*n* = 22,908). Among the countries analyzed, 69.7% (*n* = 23) experienced double exposures, though only 2.4% (*n* = 938) of unique DHS clusters experienced double exposures. Table 10 presents the results of logistic regressions for key SRH outcome variables of interest in relation to double exposures.

**Table 10:** Associations of double climate exposures with SRH attitudes and practices.

SRH outcome	Odds ratio	95% CI	SE
Modern contraception use	0.82***	(0.81–0.82)	0.03
Traditional contraception use	0.86***	(0.85–0.88)	0.01
No contraception use	1.22***	(1.18–1.27)	0.02
Short-acting contraception use	0.89***	(0.89–0.90)	0.01
Long-acting contraception use	0.81***	(0.80–0.81)	0.01
Desire for children	1.30***	(1.25–1.34)	0.01
Desire for children (in the next 2 years)	1.22***	(1.18–1.37)	0.02
Desire for children (in more than 2 years)	0.97	(0.94–1.00)	0.02
Fertility decisiveness	1.08***	(1.07–1.09)	0.01
Contraceptive autonomy: solo decision-making	1.07***	(1.06–1.08)	0.01
Contraceptive autonomy: joint decision-making	0.99***	(0.98–1.00)	0.02

Double exposure refers to experiencing both extreme temperature (tmax_extreme_z>1) and extreme precipitation (precip_extreme_z>1) within the previous year, as opposed to no exposure (neither extreme) or single exposure (only one climate extreme). Modern and traditional contraception use models are multinomial logit models with the reference group “No Contraception Use.” Short-acting and long-acting contraception models are multinomial logit models with reference group “No Contraception Use.” Contraceptive autonomy models are multinomial logit models with reference group “Husband Decides.” All other models are binary logistic regressions.

*p < .05.

**p < .01.

*** *p* < .001.

The findings indicate that double exposures were significantly associated with contraception usage behaviors: 18% lower odds of reporting modern contraception use (OR: 0.82; *P* < 0.001), 14% lower odds of reporting traditional contraception use (OR: 0.86; *P* < 0.001), and 22% higher odds of not reporting any contraception use (OR: 1.22; *P* < 0.001). With respect to type of modern contraception used, double exposures were associated with 11% lower odds of reporting use of short-acting contraception (OR: 0.89; *P* < 0.001) and 19% lower odds of reporting use of long-acting contraception (OR: 0.81; *P* < 0.001). Double exposures were associated with 22% higher odds of reporting the desire to have children (OR: 1.22; *P* < 0.001). Finally, double exposures were associated with 7% higher odds of the respondent being the sole decisionmaker over contraception use (contraceptive autonomy) (OR: 1.07; *P* < 0.001) relative to their husband.

## 4 Discussion

Climate projections suggest that around the world, individuals will be increasingly exposed to climate anomalies by the end of the century, with growing evidence suggesting that exposure to climate change is associated with adverse effects on SRH (20, 43, 44). The findings presented in this paper suggest that exposure to extreme heat and precipitation—considered both independently and jointly—likely impact women's reproductive attitudes and practices, including contraception use, fertility preferences, and contraceptive autonomy. Across the pooled sample, exposure to both extreme heat and extreme precipitation was associated with higher odds of non-use of contraception and lower odds of desire for children. Although double exposures were rare in our analysis, they were associated with lower odds of modern contraception use and higher odds of desire for children. Sociodemographic analyses also highlight the vulnerabilities that younger, unmarried, and childless women—as well as those living in poverty, without education, and in rural areas—may experience in accessing and using contraception when faced with climate extremes.

Our results suggest the associations between exposure to climate anomalies and contraception use, fertility preferences, and contraceptive autonomy are contingent on various sociodemographic intersections (e.g., reproductive parity, age, marital status, education, wealth etc.), geography (e.g., country, urban/rural residence, etc.), and exposure (e.g., type of exposure, scope of exposure, and co-occurrence of exposures). These findings are consistent with previous research which has found divergent effects of temperature and precipitation anomalies on reproductive attitudes, practices, and outcomes (28, 38, 45), as well as divergent effects on sexual activity (46–48). We build on prior work examining connections between climate variability and fertility preferences (38, 45) by integrating contraception use, which may mediate the relationship between exposure to anomalies and subsequent fertility attitudes, practices, and outcomes; we also build on prior work examining connections between drought, contraception use, and fertility preferences (28) to consider additional climate extremes, namely, temperature and precipitation anomalies.

The modest but significant effects in our pooled analysis demonstrate the persistence of these relationships between exposure to climate anomalies and SRH dynamics cross-nationally; some pooled results show more substantial magnitudes, such as the observed 6% higher odds of reporting contraceptive autonomy among those exposed to temperature anomalies. Given the population-scale implications of these results, even seemingly modest effects in the pooled analyses could be substantively meaningful. For example, in a region with 10 million reproductive-age women at similar baseline contraceptive autonomy prevalence to our full sample (i.e., 15%), an odds ratio of 1.02, when converted to probabilities, would correspond to an approximate 0.3 percentage point increase in contraceptive autonomy from 15.0% to about 15.3%. While this shift may seem small for any single individual, when scaled to a regional population of 10 million reproductive-age women with the same baseline prevalence, this effect would translate to about 30,000 additional women experiencing contraceptive autonomy. However, the variability observed in more focused analyses targeting geographic and sociodemographic factors suggests that pooled models may obscure important trends and vulnerabilities among specific sub-groups, and that future research should prioritize more nuanced analyses to most accurately capture variations in SRH attitudes, preferences, and outcomes.

Many of the country-specific analyses revealed stronger relationships than those observed in the pooled analyses. It is not clear from this dataset what factors drive this variation; country-level differences in climate resilience, gender equity, health policy, health service delivery, economy and resource availability, environment, migration and displacement, conflict and instability, cultural practices, and other factors can influence observed differences in the country-level analyses, but such indicators are not captured in DHS datasets. Ultimately, there is a need for research methodologies that use expanded quantitative and qualitative datasets to ground-truth analyses, contextualize findings, and identify and interpret trends and patterns across geographies.

When examining trends among sociodemographic sub-groups, the association between climate exposure and SRH outcome variables of interest varied by climate exposure, SRH outcome variable, and sociodemographic sub-group. Notably, when exposed to either extreme heat or extreme precipitation, respondents without children demonstrated lower odds of modern contraception use and lower odds of desire for children. Moreover, young women demonstrated higher odds of modern contraception use associated with exposure to extreme heat but lower odds of modern contraception use associated with exposure to extreme precipitation. These findings suggest the importance of climate-responsive programming targeted to these specific groups' needs. These findings illustrate that reproductive health attitudes and practices are personal, conditional, and complex, and thus should not be approached with one-size-fits all strategies. The results also highlight the importance of rights-based, contextually adapted SRH service delivery that is agile and responsive to both changing climate hazards and shifts in preferences around fertility and contraceptive methods among different demographic groups and across different geographies. Such efforts would be complemented by research that explores how climate anomalies and different aspects of identity interact with changing preferences for reproductive health, including contraceptive method choice, over time; this research could enhance gender-transformative social and behavior change programming to support informed decision-making, autonomy, equity, and accessibility of rights-based services.

Importantly, double exposures were associated with overall lower odds of reporting use of either modern or traditional contraception and higher odds of reporting desire for children. This is consistent with findings that physical/weather disasters may be associated with increased fertility rates, which may be driven by psychological factors and behavioral mechanisms including child replacement and partnership dynamics (49). However, these trends are not universal, and could also be affected by socioeconomic effects and restricted reproductive health service access (50). Moreover, if exposure to climate hazards—and associated impacts on health, the economy, food security, and other aspects of society—increases in frequency and/or severity, the trends observed in this and other similar studies could shift in the future. For example, while infrequent exposure to climate hazards might increase desire for children, chronic or recurrent exposure could strain resources for climate resilience and thus, make individuals less willing to expand their families. In addition, aggregate pro-fertility trends (e.g., trends suggesting greater desire for children and lower use of modern conception) do not negate the need for high-quality, accessible SRH services in all contexts to ensure that all people have full autonomy over their reproductive health decisions, including access to information, access to their contraceptive technologies of choices, and the ability to make decisions about whether and when to have children. Future research priorities include examining additional strategies for operationalizing double exposure definitions, exploring the impact of double exposures over a larger time horizon, and developing multiple exposure measures that integrate additional climate hazards.

Our results also draw attention to the risks to contraceptive products associated with extended extreme heat exposure. Of the women in our study who used heat-sensitive contraception methods, over 41% lived in regions where the average temperature exceeded storage stability thresholds. Climate change associated events like extreme heat and extreme weather are associated with a range of adverse pregnancy and neonatal outcomes (20, 51, 52), raising the stakes for unplanned pregnancies that may occur as a result of contraceptive failure. It is possible that some methods—such as those stored at home, like condoms, oral contraception pills, and self-administered injectables—may face more actual exposure above storage thresholds than others. However, health facilities, storage warehouses, transportation infrastructure, and pharmacies in LMICs are also vulnerable to extreme heat; they may experience power blackouts or lack electric or cooling systems altogether. Therefore, further research to evaluate the risks to efficacy of each of these commodities associated with extreme heat exposure, including research that evaluates thresholds for product degradation, integrates additional exposure metrics such as humidity and wet bulb temperature, develops innovative heat-adapted contraceptive technologies, identifies the most heat-sensitive steps in the commodity delivery pathway, and tests strategies for adjusting supply chains, storage protocols, and packaging, will be important.

### 4.1 Limitations

Some limitations to the present analysis limit the strength of our conclusions but suggest pathways for future research directions. First, our study used cross-sectional DHS data to examine the association between anomaly exposure and SRH attitudes and practices. However, this approach has limitations. The correlative nature of this work precludes causal analysis. The analysis does not account for temporal shifts in attitudes and behaviors, including those that may occur as individuals experience prolonged or repeated exposure to environmental stressors. That is, specific types of beliefs or behaviors may change on different timescales in response to anomaly exposure, and repeated exposure may change the time lag over which such shifts manifest. Unfortunately, it is not possible to examine trends for individual women over time using DHS data. Additionally, our analysis is limited in its capacity to explore the complex linkages among dependent variables and how they may interact with other factors (e.g., conflict and instability, migration, displacement, crop loss, income loss, etc.) to mediate and shape reproductive health goals and outcomes.

Second, DHS data is also limited in the types of SRH data it captures. In addition to making it difficult to explore a broader suite of interactions, this also makes it difficult to evaluate other critical SRH indicators such as maternal mortality, severe maternal morbidity, safe abortion access, reproductive cancers, or comprehensive sexuality education. By excluding pregnant women from our dataset, we also excluded any women who may be pregnant due to contraceptive failure; this also may mean that our sample includes fewer women who desire children. Our findings may be subject to confounding, given the possibility of omitted variable bias (e.g., exclusion of other factors that may explain the relationship between anomalies and SRH attitudes and practices). It is possible that the true effect of climate change on reproductive attitudes and practices is much larger than presented here due to the difficulty of identifying appropriate variables and variable interactions to capture such effects.

Third, our analysis pooled multiple survey years per country (24 of 33 countries had multiple survey waves included), which averages out within-country idiosyncratic single-year effects. Additionally, pooling is helpful to achieve sufficient statistical power because exposure to climate anomalies is rare by definition (e.g., double exposures occurred for only 2.7% of respondents within our sample). However, pooling raises concerns over temporal heterogeneity—specifically, the potential masking of within-country variation in climate-SRH associations over time. To address this limitation, we conducted a sensitivity analysis comparing pooled country-level odds ratios with those from each country's most recent survey wave of the subset of countries with multiple country years in the sample. Results of the sensitivity analysis (available in Supplementary Material 3.3) indicated pooling likely provides reliable estimates of core climate-SRH relationships without the introduction of substantial bias. With that said, modeling country-specific temporal dynamics represents an area of value for future research; computational considerations and limits in our already parameter-heavy models with 33 country fixed effects, 17 year fixed effects, and 36 region × month of interview interactions precluded the inclusion of country-year fixed effects. Finally, our approach focused on the number of temperature and precipitation anomalies experienced by DHS respondents in the year prior to the survey. We did not consider the temporal clustering or simultaneity of anomaly events—such as whether respondents were exposed to heat waves (i.e., consecutive days of extreme heat) or whether respondents were exposed to simultaneous precipitation and temperature anomalies in the preceding year. Additionally, our measurement of precipitation anomalies evaluated exposure to extreme precipitation events and thus precluded an analysis of the association of prolonged dry spells or drought with SRH outcomes; we also did not consider other meteorological and environmental data such as humidity, wet bulb temperature, or soil moisture. Those interested in the association between drought and contraception use and contraceptive autonomy may wish to review the work of Somefun et al. (28).

Ultimately, expanded analyses of various measures of exposure to climate hazards and SRH attitudes, practices, and outcomes would provide a more nuanced understanding of how climate change may affect SRH. Future research could also explore the use of longitudinal datasets and utilize mixed-methods approaches to complement quantitative analyses with qualitative data to explore additional domains of SRH and provide additional detail about how climate anomaly exposure is related to SRH attitudes, practices, and outcomes over time.

### 4.2 Future implications

Despite growing evidence that climate change poses an urgent threat to SRH, the impact of climate change on contraceptive services has been understudied (20). This study contributes to addressing this urgent challenge by expanding the knowledge base around the relationship between exposure to climate anomalies and contraceptive use, fertility preference, and contraceptive autonomy. Understanding the complexities of how historic and future exposures to climate extremes may relate to reproductive attitudes and practices, particularly considering nuances around types of exposures, demographic characteristics, and geography, is critical for developing evidence-based policy and programming aimed at ensuring effective and climate-resilient SRH service delivery that provides responsive and rights-based care. To date, there have been significant challenges in making progress towards meeting the 1.5°C warming target set out in the Paris Agreement; as a result, the effects of climate change are increasingly felt by populations across the globe—especially those in LMICs. To meet these growing climate adaptation needs, detailed and robust quantitative studies are urgently required to explore the complex and compounding effects of exposure to diverse climate hazards on a range of SRH domains. These data can be used to ensure effective programming and direct limited resources to populations at highest risk. More broadly, targeted research to inform climate-smart SRH interventions will be vital for both building resilience to climate change and preserving and continuing progress towards achieving gender equity and sexual and reproductive health and rights for all.

## Data Availability

The original contributions presented in the study are included in the article/Supplementary Material, further inquiries can be directed to the corresponding author.
