# Supplementary material for: The complex relationship between climate anomalies and reproductive attitudes and practices in low- and middle-income countries
Source: Front Glob Womens Health. 2025 Sep 16;6:1548648. doi: 10.3389/fgwh.2025.1548648 (PMC12482920; doi:10.3389/fgwh.2025.1548648)
Supplement: Supplementary file 1 [file Supplementaryfile1.docx]

Supplementary Material

# Recoding Strategy

**Recoding strategy for variables used in this analysis:**

*Definitions below from the variable description subtab for each respective variable on the IPUMS DHS website and edited as needed for clarity, accessible at* [*https://www.idhsdata.org*](https://www.idhsdata.org)*.*

**Any Contraception**: FPMETHNOW (V312 - “Current method of FP”) reports the contraceptive method the woman is currently using. When children are chosen as the unit of analysis, the woman respondent indicates the mother of the child. Answers are grouped under the broad categories of "modern methods," "traditional methods," and "other methods," each with a different first digit. Coded as a binary variable set equal to 0 or 1 if FPMETHNOW !=000 (i.e., Not using) & FPMETHNOW<=996 (i.e., “Don’t Know; Missing; NIU responses).

**Modern Contraception**: FPMETHNOW (V312 - “Current method of FP”) reports the contraceptive method the woman is currently using. When children are chosen as the unit of analysis, the woman respondent indicates the mother of the child. Answers are grouped under the broad categories of "modern methods," "traditional methods," and "other methods," each with a different first digit. Coded as a binary variable set equal to 0 or 1 if FPMETHNOW>=1 & FPMETHNOW<=151 (i.e., Pill = 101; IUD = 102; Norplant/Implants = 103; Male Condom = 104; Female Condom = 105; Female Sterilization = 106; Male Sterilization = 107; Injections = 110; 3-Monthly Injection = 111; Monthly Injection = 112; Diaphragm/Foam/Jelly = 120; Diaphragm = 121; Diaphragm/Foam = 122; Diaphragm/Jelly = 123; Foam or Jelly = 124; Foaming tablets = 125; Vaginal methods = 126; Lactational amenorrhea (LAM) = 130; Prolonged breastfeeding = 131; Emergency contraception = 140; Other modern method = 150; Contraceptive patch = 151).

**Traditional Contraception**: FPMETHNOW (V312 - “Current method of FP”) reports the contraceptive method the woman is currently using. When children are chosen as the unit of analysis, the woman respondent indicates the mother of the child. Answers are grouped under the broad categories of "modern methods," "traditional methods," and "other methods," each with a different first digit. Coded as a binary variable set equal to 0 or 1 if FPMETHNOW>=200 & FPMETHNOW<=996 (i.e., Abstinence or periodic abstinence = 210; Periodic abstinence = 211; Cycle Beads/Standard days method = 212; Abstinence = 213; Mucus method = 214; Natural family planning (unspecified) = 215; Withdrawal = 220; Other traditional/folkloric methods = 230; Herbs/Plants = 231; Gris-Gris/Amulet = 232; Astrology = 233; Strings = 234; Other unspecified method = 300; Other specific methods 1-4 = 301-304.)

**Short-Acting Contraception**: FPMETHNOW (V312 - “Current method of FP”) reports the contraceptive method the woman is currently using. When children are chosen as the unit of analysis, the woman respondent indicates the mother of the child. Answers are grouped under the broad categories of "modern methods," "traditional methods," and "other methods," each with a different first digit. Coded as a binary variable set equal to 0 or 1 if using any short-acting contraceptive method (i.e., Pill = 101; Male Condom = 104; Female Condom = 105; Diaphragm/Foam/Jelly = 120; Diaphragm = 121; Diaphragm/Foam = 122; Diaphragm/Jelly = 123; Foam or Jelly = 124; Lactational Amenorrhea (LAM) = 130; Emergency Contraception = 140; Contraceptive Patch = 151).

**Long-Acting Contraception**: FPMETHNOW (V312 - “Current method of FP”) reports the contraceptive method the woman is currently using. When children are chosen as the unit of analysis, the woman respondent indicates the mother of the child. Answers are grouped under the broad categories of "modern methods," "traditional methods," and "other methods," each with a different first digit. Coded as a binary variable set equal to 0 or 1 if using any long-acting contraceptive method (i.e., IUD = 102; Norplant/Implants = 103; Injectable Contraception = 110; 3-Monthly Injection = 111; Monthly Injection = 112; Female Sterilization = 106; Male Sterilization = 107).

**Injectable Contraception**: FPMETHNOW (V312 - “Current method of FP”) reports the contraceptive method the woman is currently using. When children are chosen as the unit of analysis, the woman respondent indicates the mother of the child. Answers are grouped under the broad categories of "modern methods," "traditional methods," and "other methods," each with a different first digit. Coded as a binary variable set equal to 0 or 1 if using any injectable contraceptive method (i.e., General Injections = 110; 3-Monthly Injection = 111; Monthly Injection = 112).

**Abstinence Period:** FPMETHNOW (V312 - “Current method of FP”) reports the contraceptive method the woman is currently using. When children are chosen as the unit of analysis, the woman respondent indicates the mother of the child. Answers are grouped under the broad categories of "modern methods," "traditional methods," and "other methods," each with a different first digit. Coded as a binary variable set equal to 0 or 1 if using abstinence or periodic abstinence as a contraceptive method (i.e., Abstinence or periodic abstinence = 210; Periodic abstinence = 211).

**Female Sterilization:** FPMETHNOW (V312 - “Current method of FP”) reports the contraceptive method the woman is currently using. When children are chosen as the unit of analysis, the woman respondent indicates the mother of the child. Answers are grouped under the broad categories of "modern methods," "traditional methods," and "other methods," each with a different first digit. Coded as a binary variable set equal to 0 or 1 if using female sterilization as a contraceptive method (i.e., Female Sterilization = 106).

**Desire for Another Child**: KIDDESIRE (V605 - “Desire for more children”) is a constructed variable that distinguishes between women who want another child soon (in less than two years), who want another child later (in 2+ years), who want no more children, and who aren't sure (about timing or about the number of children desired). The construction of this variable has changed across phases of the DHS and varies across countries (only sometimes distinguishing between groups of women not at risk of pregnancy due to sterilization, infecundity, or virginity). To maximize comparability across samples, IPUMS-DHS employs composite coding. Coded as a binary variable set equal to 0 or 1 if the woman wants another child, whether soon (KIDDESIRE=11, within 2 years), later (KIDDESIRE=12, after 2+ years), or at an unspecified time (KIDDESIRE=13).

**Desire for Another Child in the Next Two Years**: KIDDESIRE (V605 - “Desire for more children”) is a constructed variable that distinguishes between women who want another child soon (in less than two years), who want another child later (in 2+ years), who want no more children, and who aren't sure (about timing or about the number of children desired). The construction of this variable has changed across phases of the DHS and varies across countries (only sometimes distinguishing between groups of women not at risk of pregnancy due to sterilization, infecundity, or virginity). To maximize comparability across samples, IPUMS-DHS employs composite coding. Coded as a binary variable set equal to 0 or 1 if the woman wants another child soon (i.e., KIDDESIRE=11, meaning wants a child within the next 2 years).

**Decisiveness in fertility preferences**: KIDDESIRE (V605 - “Desire for more children”) is a constructed variable that distinguishes between women who want another child soon (in less than two years), who want another child later (in 2+ years), who want no more children, and who aren't sure (about timing or about the number of children desired). The construction of this variable has changed across phases of the DHS and varies across countries (only sometimes distinguishing between groups of women not at risk of pregnancy due to sterilization, infecundity, or virginity). To maximize comparability across samples, IPUMS-DHS employs composite coding. Coded as a binary variable set equal to 0 or 1 if the woman has clear, decisive fertility preferences (i.e., KIDDESIRE=11: wants a child within 2 years; KIDDESIRE=12: wants a child after 2+ years; KIDDESIRE=40: wants no more children; KIDDESIRE=60: unable to have more children/sterilized).

**Contraceptive Autonomy – Solo Choice**: For women using family planning, FPDECIDER (V632 - “Decision-maker for using FP”) reports who decided on the use of contraception (mainly the woman, mainly her partner, joint decision, or someone else). Coded as a binary variable set equal to 0 or 1 if the woman made the contraceptive decision by herself (i.e., FPDECIDER=1, meaning the woman was the main/sole decision-maker about using family planning).

**Contraceptive Autonomy – Joint Choice**: For women using family planning, FPDECIDER (V632 - “Decision-maker for using FP”) reports who decided on the use of contraception (mainly the woman, mainly her partner, joint decision, or someone else). Coded as a binary variable set equal to 0 or 1 if the contraceptive decision was made jointly by the woman and her partner (i.e., FPDECIDER=2, meaning it was a shared decision between both partners about using family planning).

**Contraceptive Autonomy – Husband Choice**: For women using family planning, FPDECIDER (V632 - “Decision-maker for using FP”) reports who decided on the use of contraception (mainly the woman, mainly her partner, joint decision, or someone else). Coded as a binary variable set equal to 0 or 1 if the husband/partner made the contraceptive decision alone (i.e., FPDECIDER=0, meaning the woman's partner was the main/sole decision-maker about using family planning).

**Age** (V012 - “Age”). Asked using the question, "How old were you at your last birthday?”. Responses were numeric and only included respondents between ages 15-49.

**Education** (V106 - "Educational attainment"): Asked using the question "What is the highest level of school you have attended?". Responses are typically categorized as no education, primary, secondary, or higher.

**Marital Status** (V501 - "Current marital status"): Asked using the question "What is your current marital status?". Responses typically categorized as never married, currently married, living together, widowed, divorced, or separated.

**Parity** (V201 - "Total children ever born"): Derived from birth history questions asking "In what month and year was (NAME) born?" for each child. Responses are numeric counts of total births.

**Urban Status** (V025 - "Type of place of residence"): Not directly asked but coded based on country-specific definitions of urban and rural areas. Responses are binary: urban or rural.

**Wealth Index** (V190/V191 - "Wealth index"/"Wealth index factor score"): Not directly asked but constructed from household assets and characteristics survey questions. V190 provides quintile categories from poorest to richest, while V191 provides the continuous wealth index score.

# Respondents by Country

# SM Table 2.1. *Number of respondents within each sampled country*

| **Country** | **Years** | **Total N** | **Modern Contra-ception** | **Short-Acting Contra-ception** | **Long-Acting Contra-ception** | **Desire for Children** | **Contra-ceptive Auto-nomy** |
| --- | --- | --- | --- | --- | --- | --- | --- |
| Angola | 2015 | 12,689 | 1293 (10.2%) | 952 (7.5%) | 321 (2.5%) | 6901 (54.4%) | 198 (1.6%) |
| Bangladesh | 2011, 2014 | 31,271 | 16773 (53.6%) | 10368 (33.2%) | 6405 (20.5%) | 8302 (26.5%) | 2648 (8.5%) |
| Benin | 2001, 2011 | 20,169 | 1865 (9.2%) | 1056 (5.2%) | 660 (3.3%) | 12694 (62.9%) | 825 (4.1%) |
| Burkina Faso | 2003, 2010 | 25,255 | 3632 (14.4%) | 1770 (7%) | 1859 (7.4%) | 16572 (65.6%) | 975 (3.9%) |
| Burundi | 2010, 2016 | 17,460 | 2372 (13.6%) | 486 (2.8%) | 1885 (10.8%) | 11781 (67.5%) | 246 (1.4%) |
| Cameroon | 2004, 2011 | 22,301 | 3726 (16.7%) | 2940 (13.2%) | 786 (3.5%) | 16161 (72.5%) | 1181 (5.3%) |
| Chad | 2014 | 15,122 | 623 (4.1%) | 237 (1.6%) | 384 (2.5%) | 10982 (72.6%) | 108 (0.7%) |
| Congo Democratic Republic | 2007, 2013 | 23,467 | 1889 (8%) | 1403 (6%) | 431 (1.8%) | 16374 (69.8%) | 824 (3.5%) |
| Cote d'Ivoire | 2011 | 8,616 | 1309 (15.2%) | 1078 (12.5%) | 207 (2.4%) | 6588 (76.5%) | 401 (4.7%) |
| Egypt | 2005, 2008, 2014 | 46,663 | 27635 (59.2%) | 7022 (15%) | 19814 (42.5%) | 13021 (27.9%) | 4641 (9.9%) |
| Eswatini | 2006 | 4,484 | 1755 (39.1%) | 988 (22%) | 767 (17.1%) | 1836 (40.9%) | 141 (3.1%) |
| Ethiopia | 2000, 2005 | 25,904 | 2519 (9.7%) | 935 (3.6%) | 1584 (6.1%) | 15970 (61.7%) | 500 (1.9%) |
| Ghana | 2003, 2008, 2014 | 17,985 | 3156 (17.5%) | 1347 (7.5%) | 1779 (9.9%) | 11709 (65.1%) | 640 (3.6%) |
| Guinea | 2005, 2012 | 14,475 | 1052 (7.3%) | 792 (5.5%) | 256 (1.8%) | 10935 (75.5%) | 495 (3.4%) |
| Jordan | 2002, 2007, 2012 | 23,694 | 10389 (43.8%) | 3883 (16.4%) | 6464 (27.3%) | 9827 (41.5%) | 2358 (10%) |
| Kenya | 2003, 2008, 2014 | 42,985 | 14683 (34.2%) | 3379 (7.9%) | 11291 (26.3%) | 15483 (36%) | 2447 (5.7%) |
| Lesotho | 2004, 2009, 2014 | 18,952 | 7091 (37.4%) | 3793 (20%) | 3298 (17.4%) | 8133 (42.9%) | 855 (4.5%) |
| Liberia | 2007, 2013 | 14,185 | 2492 (17.6%) | 981 (6.9%) | 1508 (10.6%) | 9210 (64.9%) | 449 (3.2%) |
| Madagascar | 2008 | 15,330 | 3737 (24.4%) | 1035 (6.8%) | 2702 (17.6%) | 9031 (58.9%) | 615 (4%) |
| Malawi | 2000, 2004, 2010, 2016 | 63,889 | 23264 (36.4%) | 3120 (4.9%) | 20141 (31.5%) | 33075 (51.8%) | 2654 (4.2%) |
| Mali | 2001, 2006, 2012 | 32,644 | 2874 (8.8%) | 1439 (4.4%) | 1431 (4.4%) | 23746 (72.7%) | 1337 (4.1%) |
| Morocco | 2003 | 15,491 | 4716 (30.4%) | 3821 (24.7%) | 895 (5.8%) | 3277 (21.2%) | 1081 (7%) |
| Mozambique | 2011 | 12,039 | 2035 (16.9%) | 1349 (11.2%) | 684 (5.7%) | 7114 (59.1%) | 398 (3.3%) |
| Namibia | 2006, 2013 | 16,694 | 8401 (50.3%) | 3924 (23.5%) | 4469 (26.8%) | 7832 (46.9%) | 1085 (6.5%) |
| Niger | 2012 | 9,616 | 1349 (14%) | 1049 (10.9%) | 298 (3.1%) | 8113 (84.4%) | 650 (6.8%) |
| Nigeria | 2003, 2008, 2013 | 69,669 | 7973 (11.4%) | 5184 (7.4%) | 2627 (3.8%) | 48290 (69.3%) | 1443 (2.1%) |
| Rwanda | 2005, 2010, 2014 | 34,899 | 7611 (21.8%) | 2011 (5.8%) | 5600 (16%) | 17588 (50.4%) | 681 (2%) |
| Senegal | 2005, 2010, 2012, 2015, 2016 | 55,796 | 6563 (11.8%) | 2117 (3.8%) | 4427 (7.9%) | 45371 (81.3%) | 1573 (2.8%) |
| Tanzania | 2010, 2015 | 19,724 | 5041 (25.6%) | 1721 (8.7%) | 3318 (16.8%) | 12279 (62.3%) | 808 (4.1%) |
| Togo | 2013 | 8,470 | 1543 (18.2%) | 624 (7.4%) | 915 (10.8%) | 5638 (66.6%) | 321 (3.8%) |
| Uganda | 2001, 2006, 2011, 2016 | 35,141 | 8565 (24.4%) | 2350 (6.7%) | 6214 (17.7%) | 21008 (59.8%) | 2465 (7%) |
| Zambia | 2007, 2013 | 20,812 | 6843 (32.9%) | 2989 (14.4%) | 3846 (18.5%) | 12945 (62.2%) | 1033 (5%) |
| Zimbabwe | 2005, 2010, 2015 | 24,855 | 11396 (45.8%) | 8045 (32.4%) | 3351 (13.5%) | 13999 (56.3%) | 1640 (6.6%) |

# Robustness Checks

## Evaluating Non-Linearities

We evaluated the merits of linear versus quadratic modeling approaches at the country-level for three of our primary outcomes of interest explored in the country-level and demographic subgroup analysis of the main text: 1) modern contraception use compared to no contraception use (i.e., multinomial model); 2) the desire to have children (i.e., binary logistic model); 3) reproductive autonomy (i.e., multinomial model).

We initially ran a series of pooled sample models with squared anomaly terms and then another set with cubed anomaly terms to evaluate questions of overfitting; while the cubic models suggested that overfitting was not present, the squared pooled sample models suggested potential non-linearities, primarily for the association between temperature anomaly exposure and modern contraception use. However, despite the statistical significance of some of the quadratic terms, we wanted to evaluate whether the presumptive non-linear associations at the pooled level represented genuine threshold effects, statistical artifacts from heterogeneous country-specific relationships, or model overfitting. To address these questions, we developed a country-by-country validation framework scale to help us discriminate between robust non-linear patterns and spurious quadratic effects.

For each country-climate-outcome variable combination, we fitted two competing logistic regression models. The linear model specified the log-odds of the SRH outcome as a function of the anomaly z-score, longer-term rolling climate patterns (i.e., mean max temperature in the year before survey administration; the rolling sum of precipitation in the year before survey administration), and demographic controls including age, education, wealth, urban residence, marital status, number of children, household size, and survey month, weighting by PERWEIGHT. The quadratic model added a squared anomaly term to capture potential non-linear relationships.

We developed a classification system for quadratic relationships based on the statistical properties of the fitted curves. U-shaped patterns (positive squared coefficients) indicated convex relationships with potential minima, while inverted U patterns (negative squared coefficients) suggested concave relationships with potential maxima or optimal ranges. For example, in the context of our SRH outcome variables, a U-shaped relationship for modern contraception use might indicate that being exposed to either considerably lower or higher volumes of extreme temperature days was associated with reductions in the use of modern contraception. We also classified each pattern based on whether turning points (i.e., the tipping point at which a relationship between a reproductive health outcome and the climate anomaly exposure switched direction) fell within the observed climate data range, distinguishing between true non-monotonic relationships and functionally monotonic relationships with varying rates of change. For example, a U-shaped pattern with a turning point at +2.5σ of the climate anomaly z-score might appear quadratic statistically but function as a simple negative linear relationship within the observed range of -2σ to +2σ [which is the typical range observed within the data], while a turning point at +0.3σ would represent a more observable threshold effect where the climate-health relationship reverses direction within commonly experienced climate conditions.

We employed multiple criteria to assess the appropriateness of quadratic versus linear models. Statistical validation included likelihood ratio tests, Akaike Information Criterion (AIC) and Bayesian Information Criterion (BIC) comparisons, assessment of quadratic term significance (p < 0.05), and pseudo-R² improvement evaluation. Practical validation considered effect sizes using interquartile range changes in the anomaly variables, turning point locations relative to observed data ranges, marginal effect calculations at key percentiles, and trade-offs between model interpretability and complexity. We developed a scoring system that prioritized linear models for the set of conditions where 1) quadratic terms were non-significant, 2) model improvements were minimal, 3) the BIC favored simpler specifications, 4) turning points fell outside data ranges, 5) quadratic effects were substantively small, or 5) pseudo-R² gains to the quadratic models were marginal.

Our analysis consisted of 190 successful model comparisons across all 33 countries, representing a 96% success rate from 198 potential combinations (33 countries × 2 climate variables × 3 outcomes). Both temperature and precipitation analyses were equally represented (95 analyses each), and outcome types were similarly distributed across modern contraception use (66 analyses), desire for children (66 analyses), and reproductive autonomy (58 analyses).

**Table 1:** *Summary of Model Recommendations*

| **Recommendation** | **N** | **%** | **Mean AIC Improvement** | **Mean BIC Improvement** | **Mean Quadratic p-value** |
| --- | --- | --- | --- | --- | --- |
| Use Linear Model | 139 | 73.2% | 2.89 | -4.58 | 0.347 |
| Use Quadratic Model | 32 | 16.8% | 23.37 | 16.15 | 0.0008 |
| Use Linear Model (Validation Failed) | 12 | 6.3% | 8.45 | 0.78 | 0.018 |
| Use Linear Model (Conservative) | 7 | 3.7% | 5.12 | -2.21 | 0.089 |

Linear models were recommended in 158 instances (83.2%), while quadratic models were preferred in 32 cases (16.8%). This bias toward linear model specifications emerged from our validation criteria rather than prior assumptions about model form based on statistical significance indicators alone from the squared terms. Table 1 demonstrates the clear statistical distinctions between cases receiving different recommendations, with quadratic models showing substantially larger AIC and BIC improvements alongside highly significant quadratic terms. The "validation failed" and "conservative" categories represent cases where the anomaly squared anomaly terms were statistically significant, but other criteria suggested linear models were more appropriate.

**Table 2:** *Geographic Distribution of Quadratic Model Recommendations*

| **Region** | **Countries with Quadratic Recommendations** | **Total Quadratic Cases** | **Most Common Outcome** |
| --- | --- | --- | --- |
| East Africa | Ethiopia, Kenya, Rwanda, Tanzania, Uganda | 11 | Modern Contraception (5 cases) |
| West Africa | Burkina Faso, Benin, Chad, Guinea, Nigeria, Senegal | 10 | Modern Contraception (5 cases) |
| Central Africa | Burundi, Congo Democratic Republic | 3 | Modern Contraception (2 cases) |
| Southern Africa | Lesotho, Madagascar, Mozambique, Namibia | 5 | Desire for Children (3 cases) |
| North Africa | Egypt | 2 | Modern Contraception, Desire for Children |
| South Asia | Bangladesh | 1 | Modern Contraception |

In our context, positive values for the AIC indicate the quadratic model outperforms the linear model, so the mean ΔAIC of +4.4 suggests quadratic models generally provided a better fit, but the mean ΔBIC of -2.4 indicates that BIC's harsher complexity penalty often favored the simpler linear models despite their slightly worse fit. Moreover, 67% of quadratic terms failed to achieve statistical significance (p ≥ 0.05), the mean pseudo-R² improvement from quadratic models was minimal (0.001), indicating that even statistically significant quadratic terms often provided little additional explanatory power, and our conservative decision framework prioritized interpretability over marginal statistical improvements. However, when quadratic models were recommended, they typically showed substantial AIC improvements and represented cases where genuine non-linear patterns were both statistically significant and practically meaningful.

**Table 3:** *Characteristics of Quadratic Pattern Types*

| **Pattern Type** | **N Cases** | **% of Total** | **Mean Turning Point (σ)** | **% with Turning Point in Range** | **Mean AIC Improvement** |
| --- | --- | --- | --- | --- | --- |
| U-shaped (turning point in data) | 78 | 41.1% | 0.31 | 100% | 5.22 |
| Inverted U (turning point in data) | 70 | 36.8% | 0.18 | 100% | 4.89 |
| Accelerating Negative (convex down) | 12 | 6.3% | -3.45 | 0% | 2.14 |
| Accelerating Positive (convex up) | 12 | 6.3% | 4.22 | 0% | 1.98 |
| Decelerating Negative (convex up) | 10 | 5.3% | 2.87 | 0% | 1.76 |
| Decelerating Positive (convex down) | 8 | 4.2% | -2.93 | 0% | 1.45 |

While the minority of cases, a smaller subset of cases demonstrated robust evidence for non-linear relationships, with Rwanda providing the strongest example of nonlinear patterns for modern contraception use (see Figure 1). Rwanda showed validated inverted-U relationships for both temperature and precipitation variables, with turning points at -0.24σ and -1.1σ, respectively, indicating optimal climate conditions near normal temperatures and slightly below-normal precipitation for contraceptive adoption. These patterns exhibited strong statistical validation (validation scores 12/12) and substantial model improvements (AIC gains of 18.2 for temperature, BIC gains of 10.8), demonstrating clear rise-then-decline dynamics where contraceptive use increased from extreme conditions toward moderate levels before declining again as conditions moved further from optimal ranges.

**Figure 1.** *Climate Tipping Points and Non-Linearities (Modern-Contraception)*


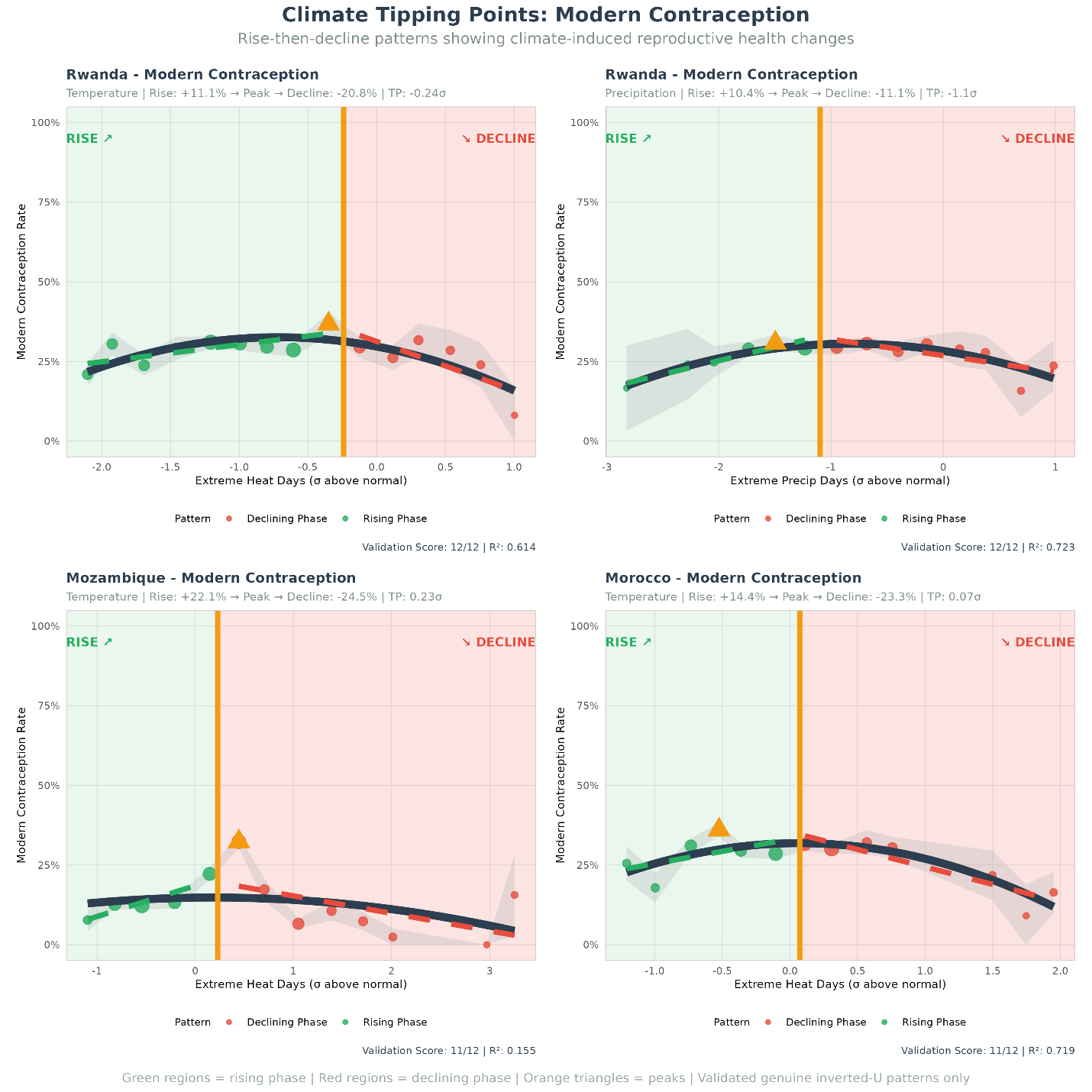


**Figure 2.** *Climate Tipping Points and Non-Linearities (Desire for Children)*


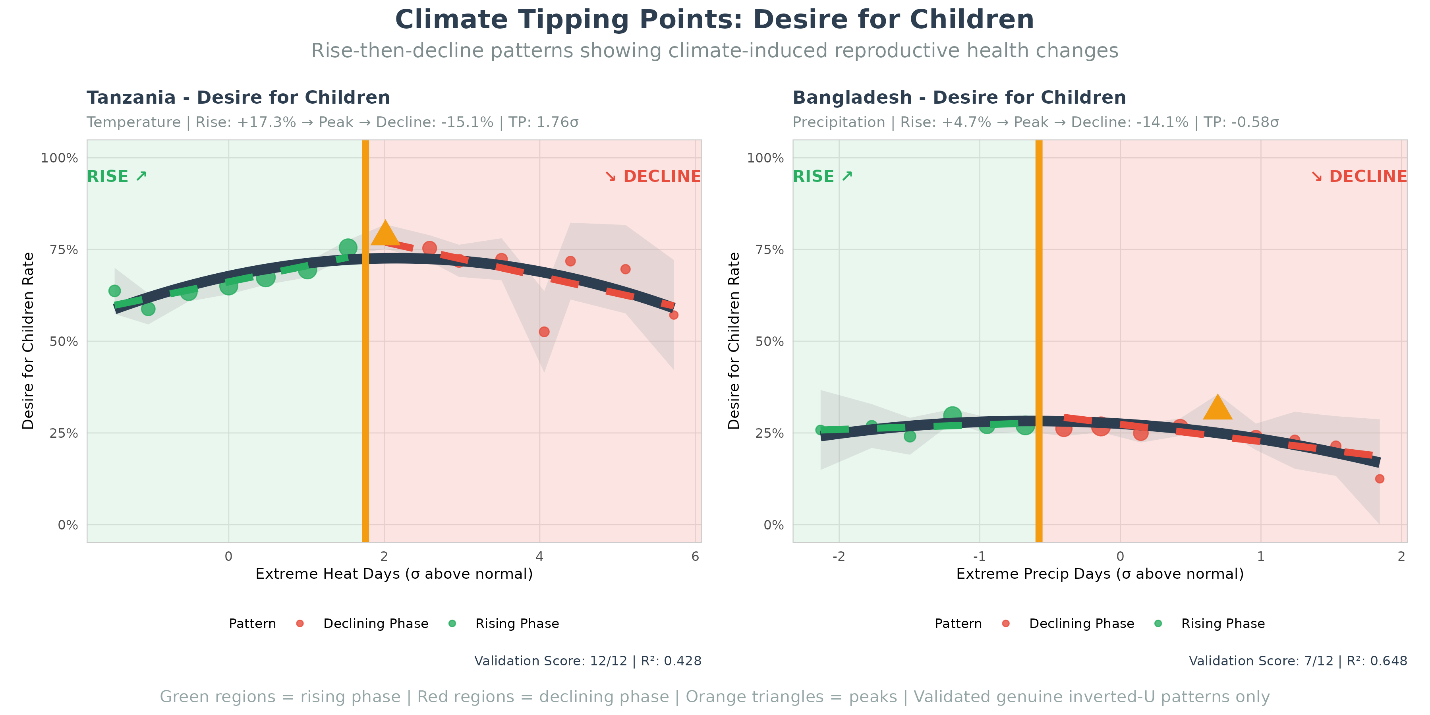


**Figure 3.** *Climate Tipping Points and Non-Linearities (Reproductive Autonomy)*


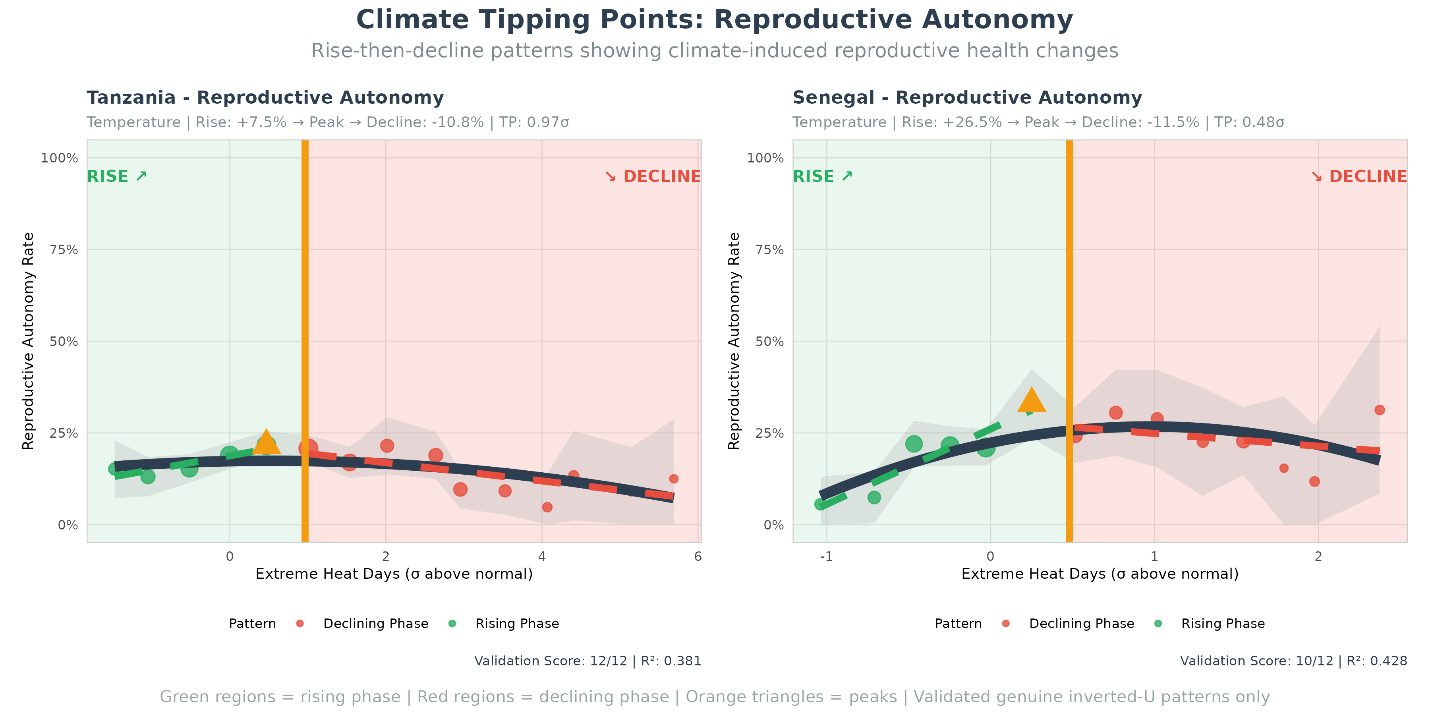


## Conditional Effects Analysis

Our results suggested heterogeneous associations between exposure to climate anomalies and the array of SRH outcomes considered. However, it is reasonable to anticipate that the effects of climate anomalies on SRH behaviors and attitudes vary across various temperature thresholds.

We sought to investigate whether variations in rolling climate values moderated the impact of exposure to climate anomalies on SRH outcomes. To achieve this, we replicated Model 3 (non-use of contraception) and Model 21 (preference for having a child within the next two years), incorporating an interaction term between the 365-day rolling average temperature and the temperature anomaly z-score. This approach enabled us to assess whether the effects of temperature anomalies on reported non-use of contraception and the preference for having a child were moderated by recent rolling mean temperatures.

Figure 3.1 illustrates the results of Models 3 and 21, modified to include the interaction term between rolling mean temperatures and z-scores. These results demonstrate how a one standard deviation change in temperature anomaly exposure differentially affects the predicted probability of non-use of contraception and the desire to have a child within the two years following survey administration across temperature thresholds at the 10th, 50th, and 90th temperature decile levels, with 99% confidence intervals.

Figure 3.1 shows significant differences in the predicted probability of preferring to have a child within the two years following survey administration at the sample mean of temperature anomaly exposure (i.e., z-score = 0). Specifically, all else equal, respondents at the 10th percentile of sample mean temperatures had a 10.5% predicted probability of desiring to have a child within the next two years, while those at the 90th percentile had an 18.1% predicted probability (P < 0.000). Additionally, the model reveals divergent effects of anomaly exposure between these two groups: exposure to temperature anomalies increased the predicted probability of preferring to have children within the next two years at the 90^th^ percentile temperature decile. However, it slightly decreased this probability at the 10^th^ percentile temperature decile.

**Figure 3.1** *How the predicted probability of desiring to have a child within the next two years and the non-use of contraception vary as a function of temperature anomaly exposure and rolling mean temperature*


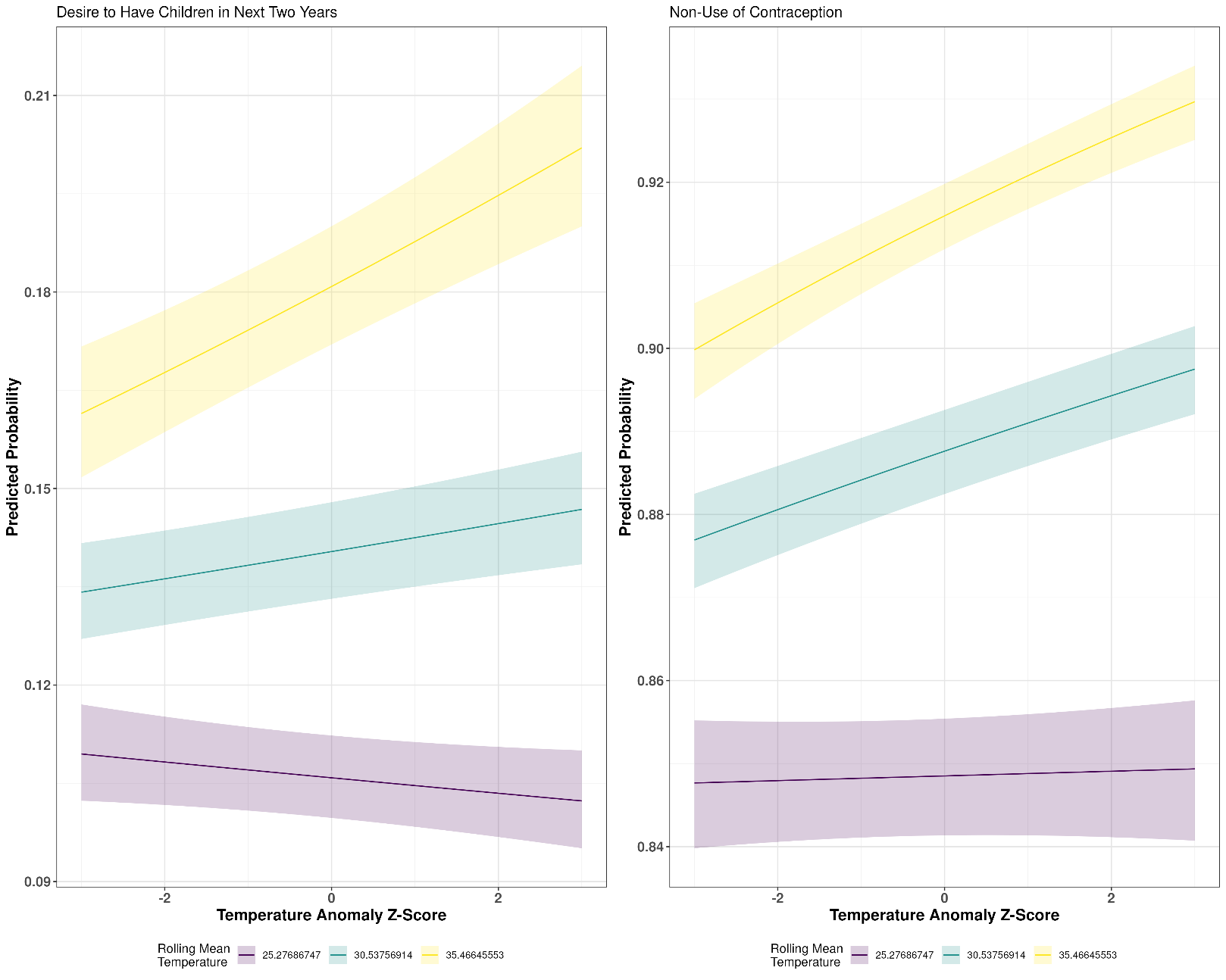


# Sociodemographic Sub-Group Analyses

We examined the effect of exposure to temperature and precipitation anomalies on various sociodemographic subgroups to evaluate the heterogeneity of the impact of anomaly exposure, presented in SM Figures 4.1-4.6. Note that the effects of precipitation anomaly exposure on modern contraceptive use and the effect of temperature anomaly exposure on fertility preference are specifically highlighted in the body of the manuscript (see Figures 3 and 4, respectively).

**SM Figure 4.1.** *Odds ratios (point estimates and 99% CI) for the effects of temperature anomaly volume on the use of modern contraceptives for various demographic groups*


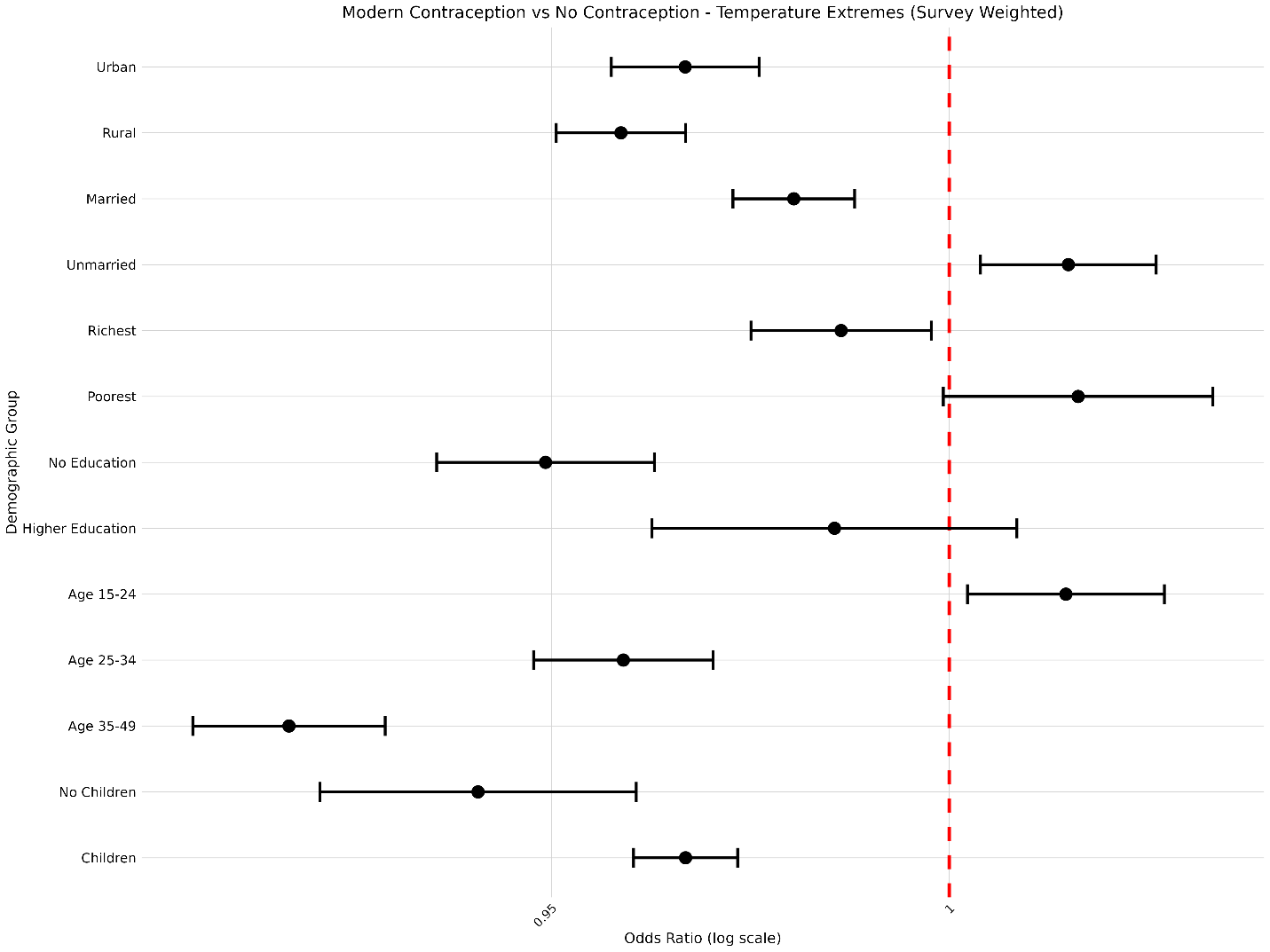


**SM Figure 4.2** *Odds ratios (point estimates and 99% CI) for the effects of precipitation anomaly volume on the use of modern contraceptives for various demographic groups*


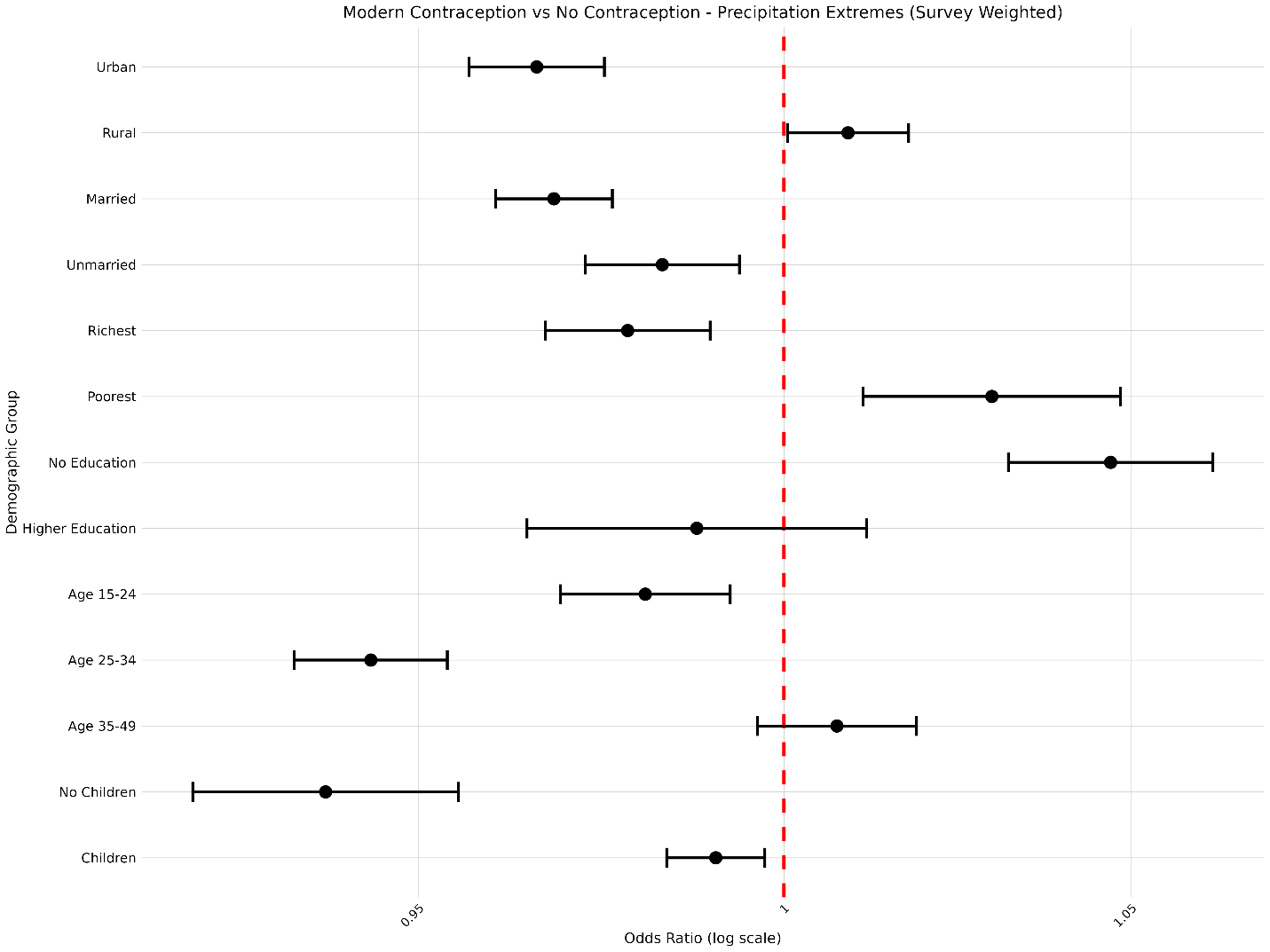


**SM Figure 4.3.** *Odds ratios (point estimates and 99% CI) for the effects of temperature anomaly volume on desire to have children for various demographic groups*


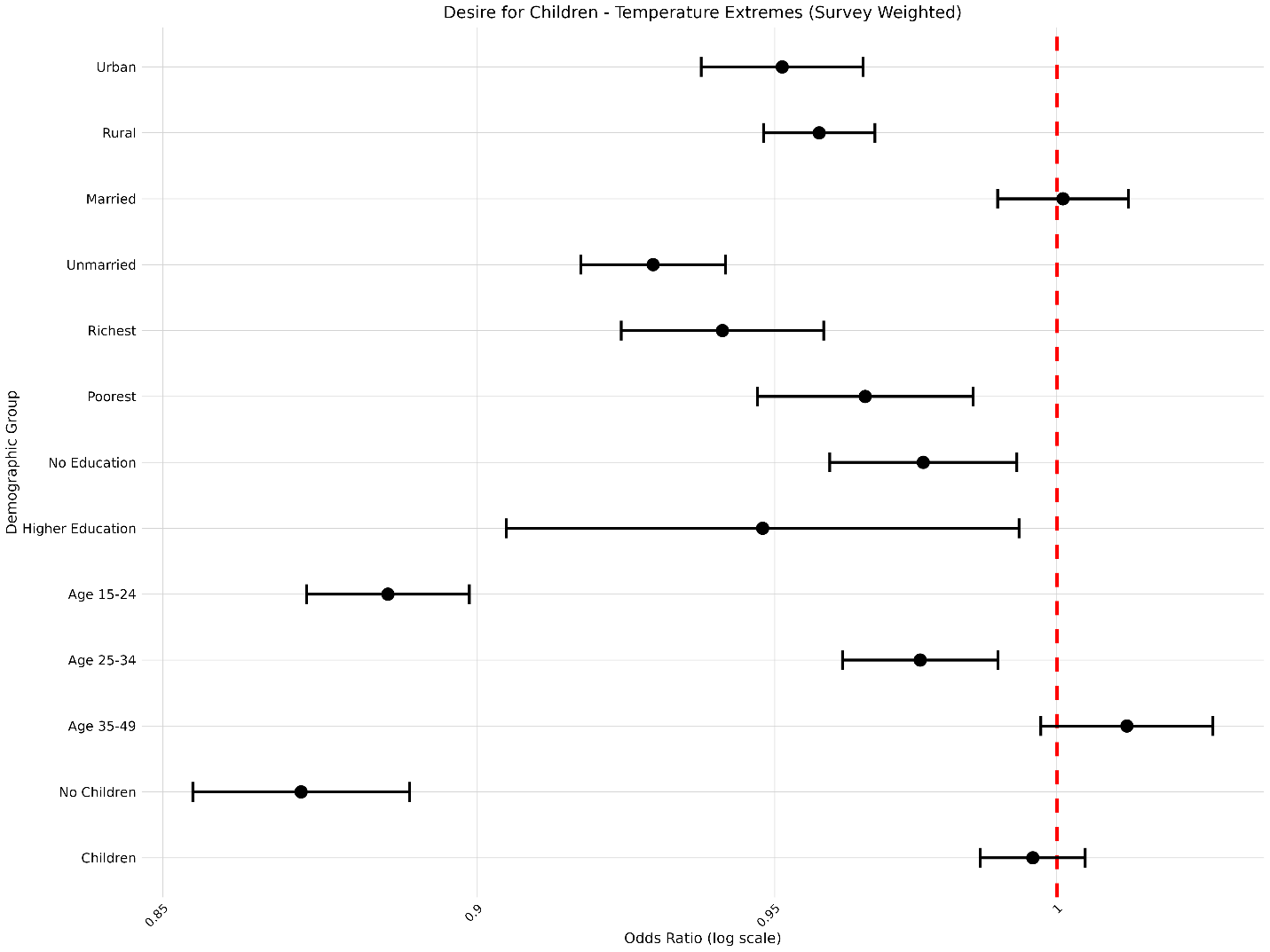


**SM Figure 4.4.** *Odds ratios (point estimates and 99% CI) for the effects of precipitation anomaly volume on the desire to have children for various demographic groups*


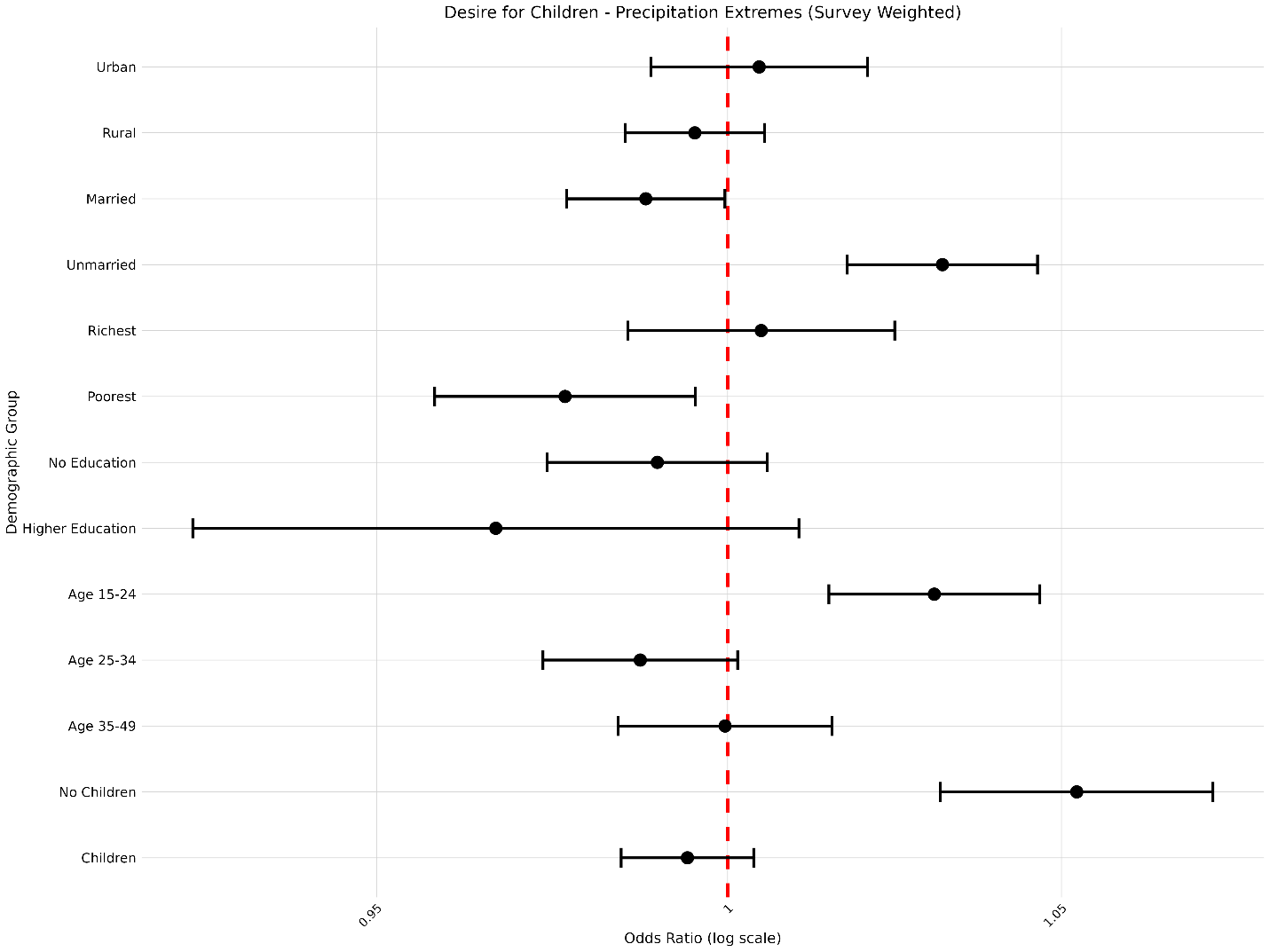


**SM Figure 4.5.** *Odds ratios (point estimates and 99% CI) for the effects of temperature anomaly volume on contraceptive autonomy for various demographic groups*


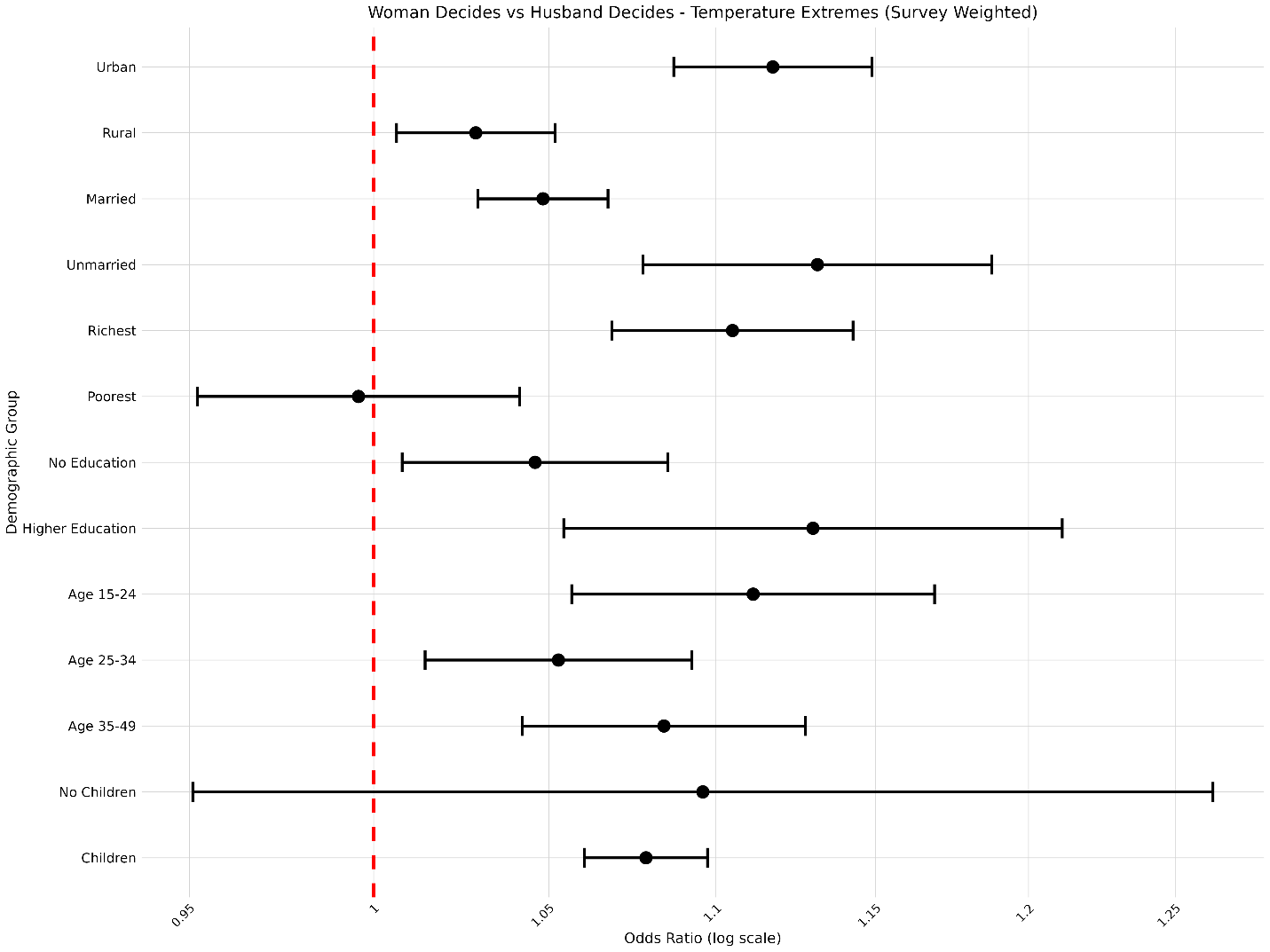


**SM Figure 4.6.** *Odds ratios (point estimates and 99% CI) for the effects of precipitation anomaly volume on contraceptive autonomy for various demographic groups*


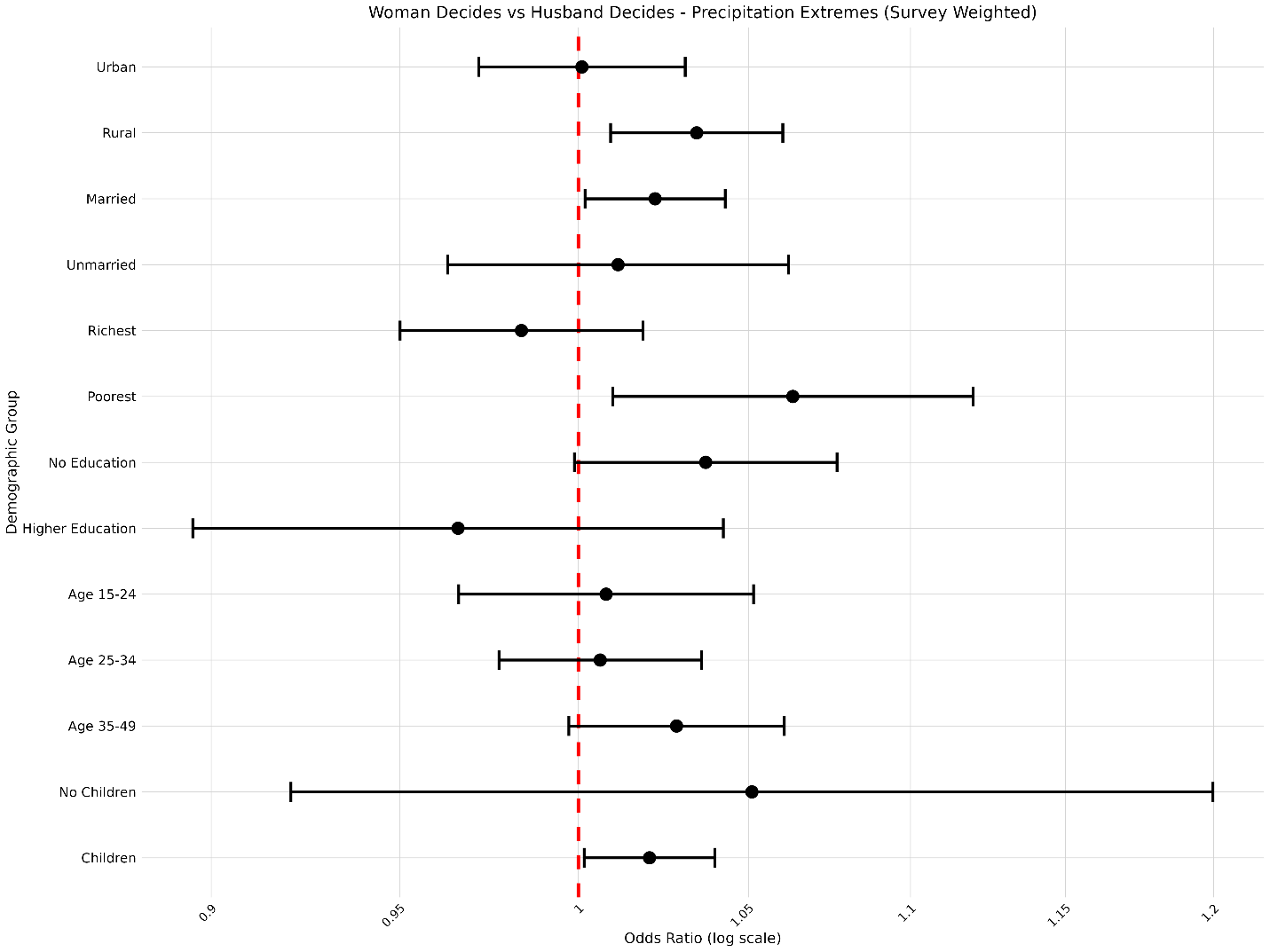


# Country-Level Analyses for Key SRH Attitudes and Practices

We examined the effect of exposure to temperature anomalies across different countries to evaluate the heterogeneity of the impact of anomaly exposure, presented in SM Table 5.1. Results are summarized in the manuscript 3.7: Country Sub-Group Analysis.

**SM Table 5.1.** *Odds Ratios by Country and SRH Outcome Variable (Temperature Anomalies)*

| **Country** | **SRH Outcome Variable** | **Odds**  **Ratio** | **Lower 95%**  **CI** | **Upper 95%**  **CI** | **p-value < 0.05** |
| --- | --- | --- | --- | --- | --- |
| Angola | Desire for Children | 0.94 | 0.91 | 0.97 | 0.00 |
| Angola | Modern Contraception | 0.96 | 0.92 | 1.00 | 0.03 |
| Angola | Solo Decision-Making | 1.10 | 0.99 | 1.21 | 0.08 |
| Bangladesh | Desire for Children | 1.13 | 1.03 | 1.24 | 0.01 |
| Bangladesh | Modern Contraception | 0.75 | 0.74 | 0.76 | 0.00 |
| Bangladesh | Solo Decision-Making | 1.21 | 1.18 | 1.24 | 0.00 |
| Benin | Desire for Children | 0.96 | 0.90 | 1.02 | 0.21 |
| Benin | Modern Contraception | 0.80 | 0.74 | 0.86 | 0.00 |
| Benin | Solo Decision-Making | 1.18 | 1.01 | 1.38 | 0.03 |
| Burkina Faso | Desire for Children | 1.04 | 0.97 | 1.12 | 0.30 |
| Burkina Faso | Modern Contraception | 1.12 | 1.05 | 1.20 | 0.00 |
| Burkina Faso | Solo Decision-Making | 0.67 | 0.58 | 0.77 | 0.00 |
| Burundi | Desire for Children | 1.26 | 1.15 | 1.38 | 0.00 |
| Burundi | Modern Contraception | 0.84 | 0.78 | 0.91 | 0.00 |
| Burundi | Solo Decision-Making | 1.25 | 0.99 | 1.59 | 0.06 |
| Cameroon | Desire for Children | 0.93 | 0.87 | 1.00 | 0.04 |
| Cameroon | Modern Contraception | 1.08 | 1.01 | 1.15 | 0.02 |
| Cameroon | Solo Decision-Making | 1.02 | 0.90 | 1.15 | 0.79 |
| Chad | Desire for Children | 1.04 | 0.96 | 1.14 | 0.35 |
| Chad | Modern Contraception | 0.57 | 0.47 | 0.69 | 0.00 |
| Chad | Solo Decision-Making | 0.87 | 0.50 | 1.51 | 0.62 |
| Congo Democratic Republic | Desire for Children | 0.95 | 0.89 | 1.01 | 0.09 |
| Congo Democratic Republic | Modern Contraception | 1.56 | 1.44 | 1.70 | 0.00 |
| Congo Democratic Republic | Solo Decision-Making | 1.12 | 0.98 | 1.28 | 0.09 |
| Cote d'Ivoire | Desire for Children | 0.94 | 0.79 | 1.12 | 0.52 |
| Cote d'Ivoire | Modern Contraception | 1.17 | 1.00 | 1.37 | 0.05 |
| Cote d'Ivoire | Solo Decision-Making | 0.65 | 0.46 | 0.93 | 0.02 |
| Egypt | Desire for Children | 1.18 | 1.11 | 1.25 | 0.00 |
| Egypt | Modern Contraception | 0.77 | 0.74 | 0.81 | 0.00 |
| Egypt | Solo Decision-Making | 0.95 | 0.89 | 1.01 | 0.12 |
| Eswatini | Desire for Children | 2.20 | 1.28 | 3.78 | 0.00 |
| Eswatini | Modern Contraception | 1.33 | 1.10 | 1.61 | 0.00 |
| Eswatini | Solo Decision-Making | 3.10 | 1.69 | 5.67 | 0.00 |
| Ethiopia | Desire for Children | 0.91 | 0.88 | 0.95 | 0.00 |
| Ethiopia | Modern Contraception | 1.04 | 0.97 | 1.10 | 0.29 |
| Ethiopia | Solo Decision-Making | 1.00 | 0.89 | 1.14 | 0.96 |
| Ghana | Desire for Children | 0.96 | 0.90 | 1.02 | 0.21 |
| Ghana | Modern Contraception | 0.96 | 0.90 | 1.03 | 0.26 |
| Ghana | Solo Decision-Making | 0.90 | 0.77 | 1.04 | 0.15 |
| Guinea | Desire for Children | 1.22 | 1.11 | 1.35 | 0.00 |
| Guinea | Modern Contraception | 0.94 | 0.84 | 1.06 | 0.31 |
| Guinea | Solo Decision-Making | 1.02 | 0.77 | 1.36 | 0.87 |
| Jordan | Desire for Children | 1.07 | 0.93 | 1.23 | 0.33 |
| Jordan | Modern Contraception | 0.99 | 0.94 | 1.05 | 0.77 |
| Jordan | Solo Decision-Making | 1.16 | 1.06 | 1.27 | 0.00 |
| Kenya | Desire for Children | 1.07 | 1.01 | 1.12 | 0.01 |
| Kenya | Modern Contraception | 0.94 | 0.91 | 0.98 | 0.00 |
| Kenya | Solo Decision-Making | 0.88 | 0.83 | 0.93 | 0.00 |
| Lesotho | Desire for Children | 0.92 | 0.85 | 0.99 | 0.02 |
| Lesotho | Modern Contraception | 1.06 | 0.98 | 1.14 | 0.13 |
| Lesotho | Solo Decision-Making | 0.74 | 0.62 | 0.88 | 0.00 |
| Liberia | Desire for Children | 0.86 | 0.79 | 0.93 | 0.00 |
| Liberia | Modern Contraception | 1.15 | 1.08 | 1.23 | 0.00 |
| Liberia | Solo Decision-Making | 1.18 | 0.98 | 1.42 | 0.07 |
| Madagascar | Desire for Children | 1.24 | 1.14 | 1.35 | 0.00 |
| Madagascar | Modern Contraception | 0.88 | 0.82 | 0.94 | 0.00 |
| Madagascar | Solo Decision-Making | 1.41 | 1.20 | 1.66 | 0.00 |
| Malawi | Desire for Children | 1.00 | 0.97 | 1.04 | 0.97 |
| Malawi | Modern Contraception | 1.06 | 1.03 | 1.10 | 0.00 |
| Malawi | Solo Decision-Making | 1.12 | 1.04 | 1.19 | 0.00 |
| Mali | Desire for Children | 0.88 | 0.81 | 0.96 | 0.00 |
| Mali | Modern Contraception | 0.99 | 0.88 | 1.11 | 0.88 |
| Mali | Solo Decision-Making | 1.19 | 0.95 | 1.49 | 0.13 |
| Morocco | Desire for Children | 0.96 | 0.84 | 1.10 | 0.54 |
| Morocco | Modern Contraception | 0.99 | 0.90 | 1.10 | 0.92 |
| Morocco | Solo Decision-Making | 1.48 | 1.27 | 1.72 | 0.00 |
| Mozambique | Desire for Children | 1.01 | 0.96 | 1.08 | 0.64 |
| Mozambique | Modern Contraception | 0.93 | 0.86 | 1.01 | 0.09 |
| Mozambique | Solo Decision-Making | 1.00 | 0.82 | 1.22 | 0.99 |
| Namibia | Desire for Children | 0.94 | 0.90 | 0.97 | 0.00 |
| Namibia | Modern Contraception | 1.05 | 1.02 | 1.09 | 0.00 |
| Namibia | Solo Decision-Making | 1.11 | 1.04 | 1.19 | 0.00 |
| Niger | Desire for Children | 1.30 | 1.08 | 1.57 | 0.01 |
| Niger | Modern Contraception | 0.67 | 0.57 | 0.78 | 0.00 |
| Niger | Solo Decision-Making | 0.83 | 0.63 | 1.10 | 0.19 |
| Nigeria | Desire for Children | 1.13 | 1.09 | 1.17 | 0.00 |
| Nigeria | Modern Contraception | 1.00 | 0.95 | 1.04 | 0.86 |
| Nigeria | Solo Decision-Making | 1.28 | 1.15 | 1.43 | 0.00 |
| Rwanda | Desire for Children | 0.99 | 0.93 | 1.06 | 0.84 |
| Rwanda | Modern Contraception | 1.05 | 0.98 | 1.13 | 0.15 |
| Rwanda | Solo Decision-Making | 1.36 | 1.13 | 1.63 | 0.00 |
| Senegal | Desire for Children | 0.97 | 0.92 | 1.01 | 0.17 |
| Senegal | Modern Contraception | 0.94 | 0.90 | 0.98 | 0.00 |
| Senegal | Solo Decision-Making | 1.02 | 0.93 | 1.11 | 0.71 |
| Tanzania | Desire for Children | 1.03 | 0.99 | 1.07 | 0.10 |
| Tanzania | Modern Contraception | 0.96 | 0.93 | 0.99 | 0.01 |
| Tanzania | Solo Decision-Making | 0.97 | 0.90 | 1.03 | 0.32 |
| Togo | Desire for Children | 1.18 | 1.02 | 1.36 | 0.03 |
| Togo | Modern Contraception | 0.73 | 0.66 | 0.80 | 0.00 |
| Togo | Solo Decision-Making | 1.01 | 0.80 | 1.27 | 0.95 |
| Uganda | Desire for Children | 1.02 | 0.99 | 1.05 | 0.24 |
| Uganda | Modern Contraception | 0.98 | 0.95 | 1.01 | 0.12 |
| Uganda | Solo Decision-Making | 1.12 | 1.06 | 1.18 | 0.00 |
| Zambia | Desire for Children | 0.88 | 0.81 | 0.96 | 0.00 |
| Zambia | Modern Contraception | 1.23 | 1.15 | 1.32 | 0.00 |
| Zambia | Solo Decision-Making | 0.89 | 0.77 | 1.03 | 0.12 |
| Zimbabwe | Desire for Children | 1.06 | 1.01 | 1.12 | 0.03 |
| Zimbabwe | Modern Contraception | 1.01 | 0.96 | 1.06 | 0.67 |
| Zimbabwe | Solo Decision-Making | 1.07 | 0.99 | 1.17 | 0.09 |

We examined the effect of exposure to precipitation anomalies across different countries to evaluate the heterogeneity of the impact of anomaly exposure, presented in SM Table 5.2. Results are summarized in the manuscript 3.7: Country Sub-Group Analysis.

**SM Table 5.2.** *Odds Ratios by Country and Outcome (Precipitation Anomalies)*

| **Country** | **Outcome** | **Odds Ratio** | **Lower 95%**  **CI** | **Upper 95%**  **CI** | **p-value < 0.05** |
| --- | --- | --- | --- | --- | --- |
| Angola | Desire for Children | 1.03 | 0.98 | 1.08 | 0.30 |
| Angola | Modern Contraception | 1.05 | 0.97 | 1.15 | 0.22 |
| Angola | Solo Decision-Making | 1.03 | 0.80 | 1.32 | 0.83 |
| Bangladesh | Desire for Children | 0.97 | 0.91 | 1.02 | 0.24 |
| Bangladesh | Modern Contraception | 1.03 | 1.00 | 1.07 | 0.06 |
| Bangladesh | Solo Decision-Making | 0.97 | 0.91 | 1.03 | 0.37 |
| Benin | Desire for Children | 0.77 | 0.71 | 0.84 | 0.00 |
| Benin | Modern Contraception | 0.95 | 0.86 | 1.05 | 0.31 |
| Benin | Solo Decision-Making | 0.82 | 0.68 | 1.00 | 0.05 |
| Burkina Faso | Desire for Children | 0.95 | 0.90 | 1.00 | 0.05 |
| Burkina Faso | Modern Contraception | 0.93 | 0.87 | 0.99 | 0.02 |
| Burkina Faso | Solo Decision-Making | 1.02 | 0.91 | 1.15 | 0.73 |
| Burundi | Desire for Children | 1.04 | 0.96 | 1.12 | 0.34 |
| Burundi | Modern Contraception | 0.98 | 0.91 | 1.05 | 0.52 |
| Burundi | Solo Decision-Making | 1.12 | 0.91 | 1.39 | 0.28 |
| Cameroon | Desire for Children | 1.09 | 1.02 | 1.16 | 0.01 |
| Cameroon | Modern Contraception | 0.91 | 0.85 | 0.97 | 0.00 |
| Cameroon | Solo Decision-Making | 1.06 | 0.95 | 1.19 | 0.30 |
| Chad | Desire for Children | 1.09 | 1.04 | 1.15 | 0.00 |
| Chad | Modern Contraception | 0.81 | 0.74 | 0.89 | 0.00 |
| Chad | Solo Decision-Making | 1.84 | 1.40 | 2.42 | 0.00 |
| Congo Democratic Republic | Desire for Children | 0.96 | 0.92 | 1.00 | 0.06 |
| Congo Democratic Republic | Modern Contraception | 1.27 | 1.20 | 1.35 | 0.00 |
| Congo Democratic Republic | Solo Decision-Making | 0.98 | 0.89 | 1.09 | 0.74 |
| Cote d'Ivoire | Desire for Children | 0.93 | 0.82 | 1.04 | 0.21 |
| Cote d'Ivoire | Modern Contraception | 0.97 | 0.87 | 1.08 | 0.57 |
| Cote d'Ivoire | Solo Decision-Making | 1.18 | 0.93 | 1.50 | 0.17 |
| Egypt | Desire for Children | 0.70 | 0.67 | 0.72 | 0.00 |
| Egypt | Modern Contraception | 1.40 | 1.37 | 1.44 | 0.00 |
| Egypt | Solo Decision-Making | 0.94 | 0.90 | 0.98 | 0.00 |
| Eswatini | Desire for Children | 0.98 | 0.83 | 1.15 | 0.77 |
| Eswatini | Modern Contraception | 1.01 | 0.88 | 1.15 | 0.91 |
| Eswatini | Solo Decision-Making | 0.81 | 0.56 | 1.17 | 0.27 |
| Ethiopia | Desire for Children | 0.92 | 0.88 | 0.95 | 0.00 |
| Ethiopia | Modern Contraception | 1.11 | 1.03 | 1.19 | 0.00 |
| Ethiopia | Solo Decision-Making | 0.90 | 0.77 | 1.05 | 0.19 |
| Ghana | Desire for Children | 0.99 | 0.93 | 1.06 | 0.83 |
| Ghana | Modern Contraception | 0.97 | 0.91 | 1.03 | 0.32 |
| Ghana | Solo Decision-Making | 1.07 | 0.92 | 1.24 | 0.40 |
| Guinea | Desire for Children | 0.98 | 0.89 | 1.09 | 0.76 |
| Guinea | Modern Contraception | 0.77 | 0.68 | 0.86 | 0.00 |
| Guinea | Solo Decision-Making | 1.20 | 0.91 | 1.59 | 0.19 |
| Jordan | Desire for Children | 1.03 | 0.96 | 1.10 | 0.46 |
| Jordan | Modern Contraception | 0.97 | 0.92 | 1.02 | 0.19 |
| Jordan | Solo Decision-Making | 0.88 | 0.81 | 0.96 | 0.00 |
| Kenya | Desire for Children | 1.02 | 0.99 | 1.06 | 0.21 |
| Kenya | Modern Contraception | 0.92 | 0.89 | 0.95 | 0.00 |
| Kenya | Solo Decision-Making | 0.83 | 0.78 | 0.89 | 0.00 |
| Lesotho | Desire for Children | 1.01 | 0.94 | 1.09 | 0.80 |
| Lesotho | Modern Contraception | 0.92 | 0.86 | 0.99 | 0.03 |
| Lesotho | Solo Decision-Making | 0.81 | 0.69 | 0.96 | 0.01 |
| Liberia | Desire for Children | 1.05 | 0.96 | 1.15 | 0.28 |
| Liberia | Modern Contraception | 0.88 | 0.81 | 0.94 | 0.00 |
| Liberia | Solo Decision-Making | 0.88 | 0.72 | 1.07 | 0.20 |
| Madagascar | Desire for Children | 1.24 | 1.15 | 1.33 | 0.00 |
| Madagascar | Modern Contraception | 1.09 | 1.02 | 1.16 | 0.01 |
| Madagascar | Solo Decision-Making | 1.21 | 1.05 | 1.39 | 0.01 |
| Malawi | Desire for Children | 0.94 | 0.92 | 0.97 | 0.00 |
| Malawi | Modern Contraception | 1.01 | 0.98 | 1.03 | 0.52 |
| Malawi | Solo Decision-Making | 0.90 | 0.85 | 0.95 | 0.00 |
| Mali | Desire for Children | 1.13 | 1.07 | 1.19 | 0.00 |
| Mali | Modern Contraception | 0.96 | 0.90 | 1.02 | 0.22 |
| Mali | Solo Decision-Making | 1.01 | 0.88 | 1.15 | 0.87 |
| Morocco | Desire for Children | 0.90 | 0.80 | 1.01 | 0.06 |
| Morocco | Modern Contraception | 1.18 | 1.08 | 1.29 | 0.00 |
| Morocco | Solo Decision-Making | 1.14 | 1.00 | 1.29 | 0.05 |
| Mozambique | Desire for Children | 0.92 | 0.86 | 0.98 | 0.01 |
| Mozambique | Modern Contraception | 1.25 | 1.14 | 1.37 | 0.00 |
| Mozambique | Solo Decision-Making | 1.08 | 0.87 | 1.35 | 0.50 |
| Namibia | Desire for Children | 0.98 | 0.93 | 1.04 | 0.60 |
| Namibia | Modern Contraception | 1.10 | 1.04 | 1.16 | 0.00 |
| Namibia | Solo Decision-Making | 1.11 | 0.98 | 1.25 | 0.10 |
| Niger | Desire for Children | 0.80 | 0.70 | 0.92 | 0.00 |
| Niger | Modern Contraception | 0.89 | 0.79 | 1.00 | 0.06 |
| Niger | Solo Decision-Making | 1.21 | 0.97 | 1.51 | 0.09 |
| Nigeria | Desire for Children | 1.07 | 1.04 | 1.10 | 0.00 |
| Nigeria | Modern Contraception | 0.97 | 0.94 | 1.01 | 0.15 |
| Nigeria | Solo Decision-Making | 1.09 | 1.00 | 1.19 | 0.05 |
| Rwanda | Desire for Children | 0.97 | 0.93 | 1.01 | 0.11 |
| Rwanda | Modern Contraception | 1.00 | 0.95 | 1.05 | 0.98 |
| Rwanda | Solo Decision-Making | 0.84 | 0.74 | 0.95 | 0.01 |
| Senegal | Desire for Children | 0.97 | 0.93 | 1.01 | 0.12 |
| Senegal | Modern Contraception | 0.95 | 0.92 | 0.98 | 0.00 |
| Senegal | Solo Decision-Making | 0.99 | 0.93 | 1.07 | 0.85 |
| Tanzania | Desire for Children | 1.02 | 0.98 | 1.06 | 0.27 |
| Tanzania | Modern Contraception | 0.80 | 0.77 | 0.83 | 0.00 |
| Tanzania | Solo Decision-Making | 0.99 | 0.91 | 1.09 | 0.85 |
| Togo | Desire for Children | 1.24 | 1.06 | 1.44 | 0.01 |
| Togo | Modern Contraception | 0.72 | 0.63 | 0.83 | 0.00 |
| Togo | Solo Decision-Making | 0.85 | 0.62 | 1.16 | 0.30 |
| Uganda | Desire for Children | 1.05 | 1.02 | 1.09 | 0.00 |
| Uganda | Modern Contraception | 0.96 | 0.93 | 0.99 | 0.01 |
| Uganda | Solo Decision-Making | 0.93 | 0.88 | 0.99 | 0.02 |
| Zambia | Desire for Children | 1.02 | 0.97 | 1.07 | 0.43 |
| Zambia | Modern Contraception | 1.08 | 1.04 | 1.12 | 0.00 |
| Zambia | Solo Decision-Making | 1.06 | 0.97 | 1.15 | 0.20 |
| Zimbabwe | Desire for Children | 0.90 | 0.85 | 0.96 | 0.00 |
| Zimbabwe | Modern Contraception | 1.15 | 1.09 | 1.21 | 0.00 |
| Zimbabwe | Solo Decision-Making | 1.02 | 0.93 | 1.12 | 0.69 |

# Table Notes

Notes for Tables 4-8: Constant, country-fixed effects, regionally-specific month of the year fixed effects, and year-fixed effects are also included in the model. Standard errors are clustered at the level of the DHS survey cluster. Reference categories for categorical variables represent the most frequent response. The reference category for Marital Status is Married. The reference category for Urban is Rural. The reference category for Education Level is None. The reference category for Wealth is Richest. The reference category for Husband Education Years is the lowest quartile of education year. *** p<.001, ** p<.01, * p<.05, + p<.1

# Figure Notes

Notes for Figures 2-3: Constant, country-fixed effects, regionally-specific month of the year fixed effects, and year-fixed effects are also included in the model. Standard errors are clustered at the level of the DHS survey cluster.
